# Dopamine-inhibited POMC^Drd2+^ neurons in the ARC acutely regulate feeding and body temperature

**DOI:** 10.1172/jci.insight.162753

**Published:** 2022-11-08

**Authors:** Isabella Gaziano, Svenja Corneliussen, Nasim Biglari, René Neuhaus, Linyan Shen, Tamara Sotelo-Hitschfeld, Paul Klemm, Lukas Steuernagel, Alain J. De Solis, Weiyi Chen, F. Thomas Wunderlich, Peter Kloppenburg, Jens C. Brüning

**Affiliations:** 1 Neuronal Control of Metabolism group, Max Planck Institute for Metabolism Research, Cologne, Germany.; 2 Center for Endocrinology, Diabetes and Preventive Medicine (CEDP), University Hospital Cologne, Cologne, Germany.; 3 Excellence Cluster on Cellular Stress Responses in Aging Associated Diseases (CECAD) and Center of Molecular Medicine Cologne (CMMC) and; 4 Institute for Zoology, Faculty of Mathematics and Natural Sciences, University of Cologne, Germany.; 5 Obesity and Cancer group, Max Planck Institute for Metabolism Research, Cologne, Germany.; 6 National Center for Diabetes Research (DZD), Neuherberg, Germany.

**Keywords:** Endocrinology, Neuroscience, Mouse models, Obesity

## Abstract

Dopamine acts on neurons in the arcuate nucleus (ARC) of the hypothalamus, which controls homeostatic feeding responses. Here we demonstrate a differential enrichment of dopamine receptor 1 (Drd1) expression in food intake–promoting agouti related peptide (AgRP)/neuropeptide Y (NPY) neurons and a large proportion of Drd2-expressing anorexigenic proopiomelanocortin (POMC) neurons. Owing to the nature of these receptors, this translates into a predominant activation of AgRP/NPY neurons upon dopamine stimulation and a larger proportion of dopamine-inhibited POMC neurons. Employing intersectional targeting of Drd2-expressing POMC neurons, we reveal that dopamine-mediated POMC neuron inhibition is Drd2 dependent and that POMC^Drd2+^ neurons exhibit differential expression of neuropeptide signaling mediators compared with the global POMC neuron population, which manifests in enhanced somatostatin responsiveness of POMC^Drd2+^ neurons. Selective chemogenetic activation of POMC^Drd2+^ neurons uncovered their ability to acutely suppress feeding and to preserve body temperature in fasted mice. Collectively, the present study provides the molecular and functional characterization of POMC^Drd2+^ neurons and aids our understanding of dopamine-dependent control of homeostatic energy-regulatory neurocircuits.

## Introduction

Neurons of the melanocortin circuitry in the arcuate nucleus (ARC) of the hypothalamus, i.e., neurons expressing orexigenic agouti related peptide (AgRP)/neuropeptide Y (NPY) or anorexigenic proopiomelanocortin (POMC), integrate peripheral and central signals to control metabolic homeostasis, such as adapting feeding, glucose homeostasis, and energy expenditure according to the energy state of the organism (1). Recent studies have started unraveling the high degree of heterogeneity within AgRP/NPY or POMC neuronal populations: subpopulations of AgRP/NPY- or POMC-expressing neurons respond differentially to hormonal cues, exhibit discrete molecular signatures, and orchestrate distinct functional outputs in feeding, energy expenditure, and temperature control (2–6).

There is an ever-increasing understanding of peripheral signals as well as neurocircuit pathways that directly control both hunger and satiety but also the dopaminergic reward system. Interestingly, various hormonal cues that regulate homeostatic feeding via the hypothalamus, such as leptin, ghrelin, glucagon-like peptide-1 (Glp1), and insulin (1), are also modulators of dopaminergic neurons in mesolimbic networks (7–10). Moreover, food deprivation intensifies dopamine-mediated reward signaling in mice, which leptin or insulin can blunt (11–13).

Reward signals substantially affect energy homeostasis and feeding: whole-body dopamine deficiency in mice is not embryonically lethal yet causes them to starve to death postnatally (14). Conversely, human obesity is associated with decreased dopamine signaling and dopamine receptor 2 (Drd2) expression in striatal regions (15), while acute loss-of-function models of Drd2 in rodents potentiate weight gain and increased consumption of palatable food (16, 17).

The interplay between ARC neurons of the melanocortin circuitry and reward signals has been largely investigated unidirectionally. Although neuronal projections from the ARC to mesolimbic networks and their functional implications have been defined at multiple levels (reviewed in ref. 18), less is known about the effect of dopamine on AgRP/NPY and POMC neurons. While dopamine receptors are expressed on POMC neurons (19), and ARC neurons positive for tyrosine hydroxylase (TH), the rate-limiting enzyme in dopamine synthesis, provide direct neuronal input onto POMC neurons (20), the functional consequences of dopamine signaling in this cell type remain to be fully elucidated. POMC neurons have been shown to have preferential response via Drd2-like signaling, whereas AgRP/NPY neurons signal through Drd1-like receptors (20). However, in vivo investigations are largely limited to the observations that obese A^y^/a mice exhibit increased Drd2 expression levels on POMC neurons (19) and that optogenetic stimulation of ARC TH neurons increases feeding in mice (20).

Taken together, there is a well-defined interdependency of homeostatic and dopamine-associated feeding regulation, and recent studies have provided considerable evidence for dopamine-dependent control of homeostatic feeding. Nonetheless, a functional definition of specific subsets of dopamine-sensitive neurons of the melanocortin circuitry remains to be established. Here, we aim at defining dopamine-inhibited or dopamine-excited subpopulations of hypothalamic neurons in the ARC and characterizing their molecular and functional organization.

## Results

### The inhibitory Drd2 is predominantly expressed in anorexigenic POMC neurons in the ARC.

**Figure 1:**
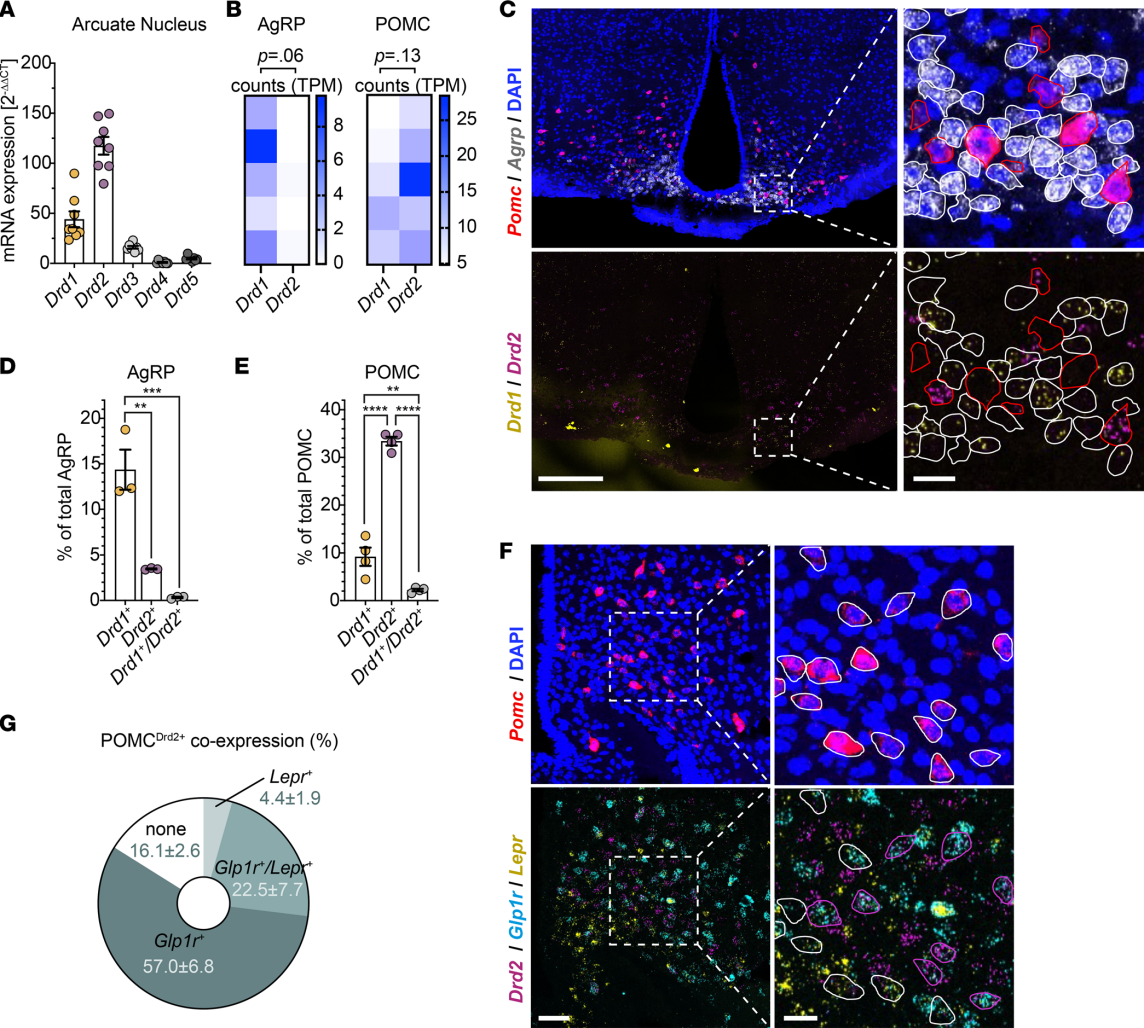
Drd1 and Drd2 colocalize on distinct neuronal populations within the ARC. (**A**) mRNA expression levels of dopamine receptor isoforms as assessed by quantitative PCR of C57BL/6N ARC micropunches. Data are represented as mean ± SEM, *n* = 8. (**B**) *Drd1* and *Drd2* mRNA expression levels in bulk RNA sequencing of AgRP/NPY or POMC neurons from random fed mice published by Henry et al. (21). Data are represented as heatmap of counts in transcripts per million per replicate. (**C**) Representative images of RNA in situ hybridization against *Pomc* and *Agrp* (top) and *Drd1* and *Drd2* (bottom) in ARC of C57BL/6N mice. AgRP/NPY and POMC neurons are indicated by white or red outlines, respectively. Scale bars: 200 μm in overviews (left), 20 μm in respective magnifications (right). (**D** and **E**) Percentage of *Drd1* and/or *Drd2* coexpressing AgRP (**D**) or POMC (**E**) neurons as quantified from RNA in situ hybridization in C57BL/6N mice (**C**). Data are represented as mean ± SEM; *n* = 3–4. (**F**) Representative images of RNA in situ hybridization against *Pomc* (top) and *Drd2*, *Glp1r*, and *Lepr* (bottom) in ARC of C57BL/6N mice. POMC^Drd2–^ neurons are outlined in white, POMC^Drd2+^ neurons in magenta. Scale bars represent 50 μm in the overviews (left) and 20 μm in the respective magnifications (right). (**G**) Percentages of POMC^Drd2+^ neurons coexpressing *Lepr* or *Glp1r* as quantified from RNA in situ hybridization in C57BL/6N mice (**F**). Data are represented as mean ± SEM; *n* = 4. (**A** and **G**) No statistical tests applied. (**B**) *P* values calculated using paired, 2-tailed Wilcoxon rank tests. (**D** and **E**) *P* values calculated using 1-way ANOVA with Tukey’s post hoc multiple comparisons test with a single pooled variance. **P < 0.01. ***P < 0.001. ****P < 0.0001.

To delineate the role and mechanisms of dopamine signaling in feeding-regulatory neurons in the ARC, we first monitored the mRNA expression of Drd subtypes in this brain region. Quantitative real-time PCR analyses on RNA isolated from micropunches from the ARC of WT mice revealed the predominant expression of *Drd1* and *Drd2* in this brain region (Figure 1A). We assessed the mRNA expression of *Drd1* and *Drd2* in the well-characterized feeding-regulatory AgRP/NPY- or POMC-expressing neurons in the ARC. Concordant with RNA-sequencing data of a previously published study (21) (Figure 1B), our RNA in situ hybridization in WT mice revealed a striking dichotomy in the ratio of *Drd1-* and *Drd2*-expressing AgRP/NPY and POMC neurons (4.1 vs. 0.3). While the proportion of *Drd1*-expressing neurons was similar between AgRP/NPY and POMC neurons (14.4% ± 2.2% vs. 9.2% ± 1.9%), a much larger proportion of anorexigenic POMC neurons specifically expressed the inhibitory *Drd2* (33.4% ± 0.9%) compared with AgRP/NPY neurons (3.5% ± 0.1%) (Figure 1, C–E).

Since our previous studies had revealed heterogenous POMC neurons characterized by differential expression of Glp1 receptor (Glp1r) and leptin receptor (Lepr) (4), we investigated whether these distinct POMC neuronal subpopulations differed in their Drd2 expression. To this end, we employed RNA in situ hybridization against *Pomc*, *Drd2*, *Glp1r*, and *Lepr*. These experiments revealed dominant expression of *Drd2* mRNA in *Glp1r*-expressing POMC neurons (57.0% ± 6.8%), while of *Lepr*-expressing POMC neurons only 4.4% ± 1.9% also expressed *Drd2* (Figure 1, F and G).

### AgRP/NPY neurons are predominantly activated by dopamine.

**Figure 2:**
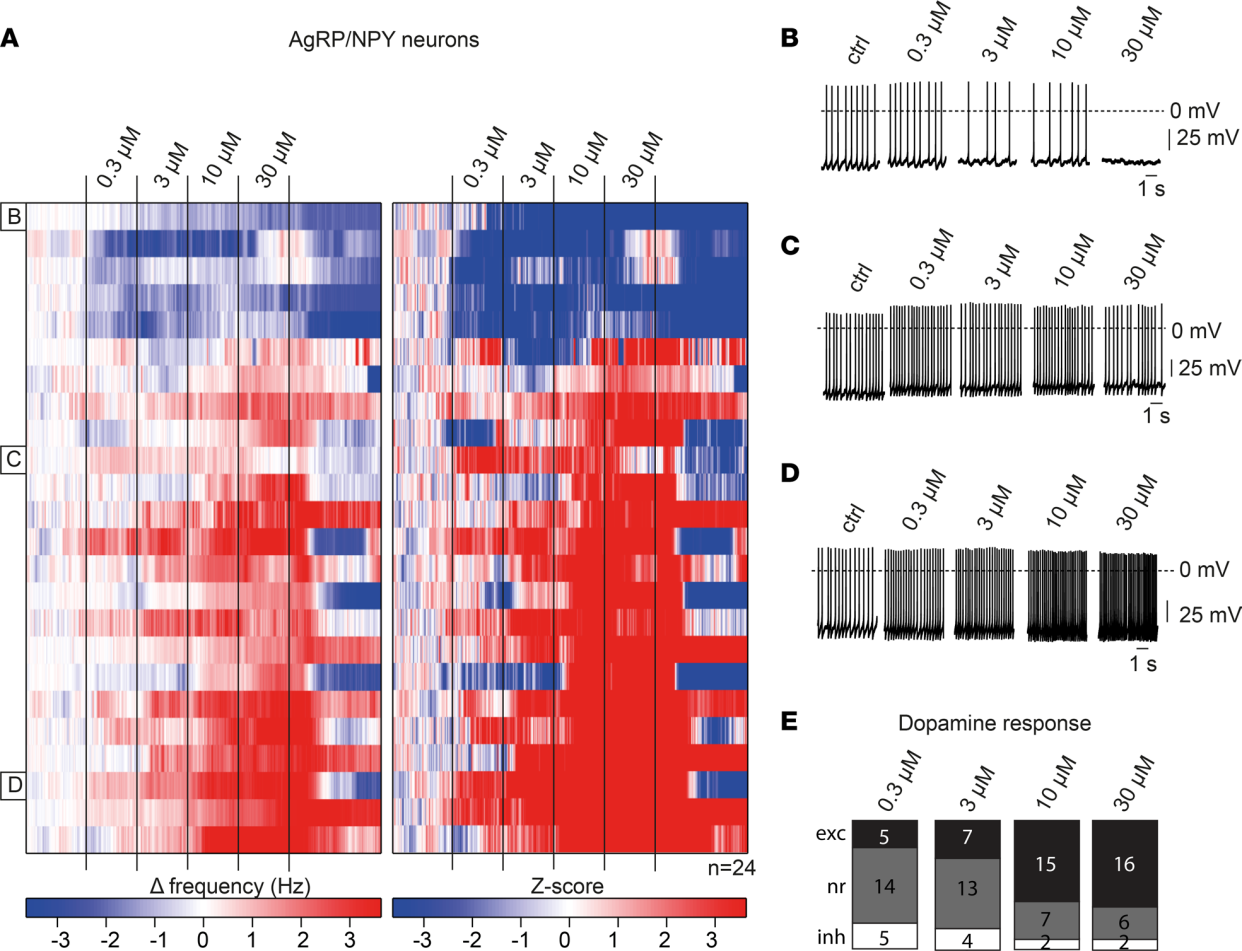
Effects of dopamine on AgRP/NPY neurons. (**A**) Heatmap of 24 perforated patch-clamp recordings of AgRP/NPY neurons in male and female NPY^GFP^ mice between 11 and 20 weeks of age. Changes in action potential frequency from baseline and corresponding *z* scores during the application of increasing dopamine concentrations (0.3 μM, 3 μM, 10 μM, 30 μM) are depicted on the left and right, respectively. (**B**–**D**) Representative original perforated patch-clamp recording of a single AgRP/NPY neuron, which was inhibited by dopamine (**B**), a neuron that weakly responded to dopamine (**C**), and a neuron that was excited by dopamine (**D**). Data are also shown as part of **A** at the indicated rows. DA, dopamine. (**E**) Statistical quantification of dopamine responses in AgRP/NPY neurons from **A** at indicated dopamine concentrations. A neuron was considered responsive if the change in firing frequency induced by drug application was 3 times larger than the SD. exc, excited; nr, not responsive; inh, inhibited.

Having identified the differential relative expression of excitatory Drd1 and inhibitory Drd2 in AgRP/NPY and POMC neurons, we aimed to assess the corresponding electrophysiological responses of AgRP/NPY neurons to dopamine. Therefore, we performed perforated patch-clamp recordings in NPY-expressing neurons in the ARC of NPY^GFP^-transgenic mice upon pharmacological blockade of glutamatergic and GABAergic synaptic inputs. Incubation with increasing concentrations of dopamine exhibited progressive reduction in action potential frequency only in few AgRP/NPY neurons (Figure 2, A and B). While other cells did not change firing frequency upon dopamine application (Figure 2, A and C), the largest proportion of NPY-expressing neurons in the ARC increased action potential frequency in a concentration-dependent manner (Figure 2, A and D). At 0.3 μM dopamine 21% of NPY neurons were significantly excited, and this proportion progressively increased, reaching more than 60% of excited neurons at 10 μM and 30 μM dopamine (Figure 2, A and E).

### Dopamine inhibits POMC neuron activity in a Drd2-dependent manner.

**Figure 3:**
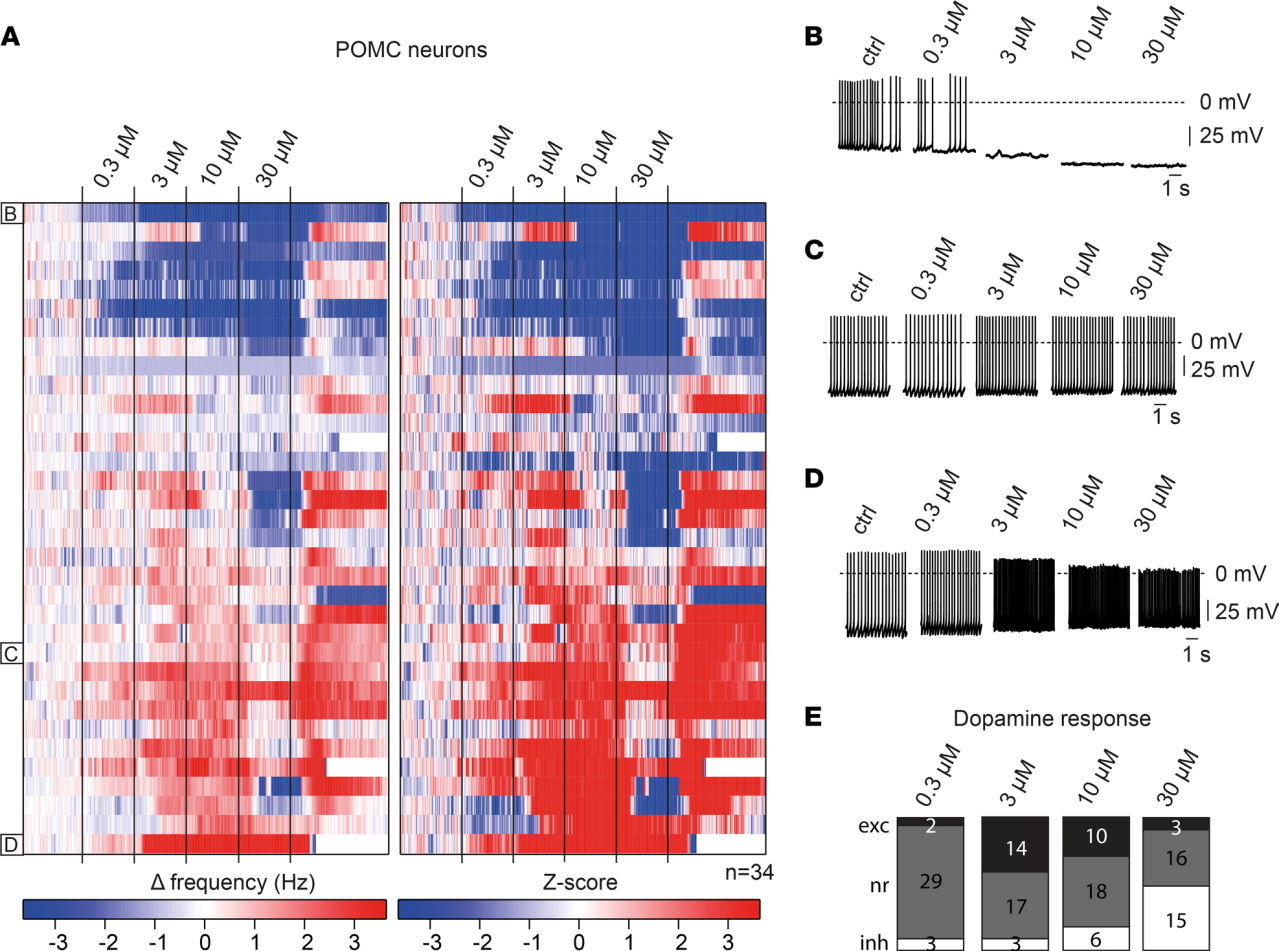
Effects of dopamine on POMC neurons. (**A**) Heatmap of 34 perforated patch-clamp recordings of POMC neurons in male and female POMC^GFP^ mice between 11 and 20 weeks of age. Changes in action potential frequency from baseline and corresponding *z* scores during the application of increasing dopamine concentrations (0.3 μM, 3 μM, 10 μM, 30 μM) are depicted on the left and right, respectively. (**B**–**D**) Representative original perforated patch-clamp recordings of a single POMC neuron inhibited by dopamine (**B**), a neuron that weakly responded to dopamine (**C**), and a neuron excited by dopamine (**D**). Data are also shown as part of **A** at the indicated rows. (**E**) Statistical quantification of dopamine responses in POMC neurons from **A** at indicated dopamine concentrations. A neuron was considered responsive if the change in firing frequency induced by drug application was 3 times larger than the SD.

We tested the responses of genetically identified POMC neurons to dopamine. Here, we performed electrophysiological patch-clamp recordings in synaptically isolated POMC neurons in the ARC of POMC^GFP^-transgenic mice. Dopamine elicited diverse response patterns in this cell type, i.e., resulting in excitation, inhibition, or no effect on action potential frequency (Figure 3, A–D, and Supplemental Figure 1A; supplemental material available online with this article; https://doi.org/10.1172/jci.insight.162753DS1). In contrast to what was observed in AgRP/NPY cells, the proportion of cells that was inhibited by dopamine was larger in POMC compared with NPY neurons (e.g., 44% vs. 8% at 30 μM dopamine), and the proportion of POMC neurons that was activated by dopamine was reduced compared with AgRP/NPY neurons (e.g., 9% vs. 67% at 30 μM dopamine) (Figure 3E and Supplemental Figure 1B). Approximately 30% of POMC neurons reduced the action potential frequency over the range of dopamine concentrations tested, in a concentration-dependent manner. A smaller proportion of POMC neurons (approximately 10%), conversely, increased the firing frequency over the range of dopamine concentrations, while about 10% did not respond significantly to dopamine application. We frequently observed high-frequency rebound firing of action potentials during washout following dopamine-induced inhibition.

Given the sex differences for POMC expression (22) and the sex-specific regulation of synaptic inputs in dopaminergic ARC neurons (23), we compared the dopamine responses in POMC neurons of male and female mice. This analysis revealed an overall similar response pattern between both sexes; however, there was a higher proportion of POMC neurons that underwent inhibition upon incubation with 10 μM dopamine in female compared with male mice (Supplemental Figure 1, A and B). These experiments point toward increased dopamine sensitivity in POMC neurons of female mice.

**Figure 4:**
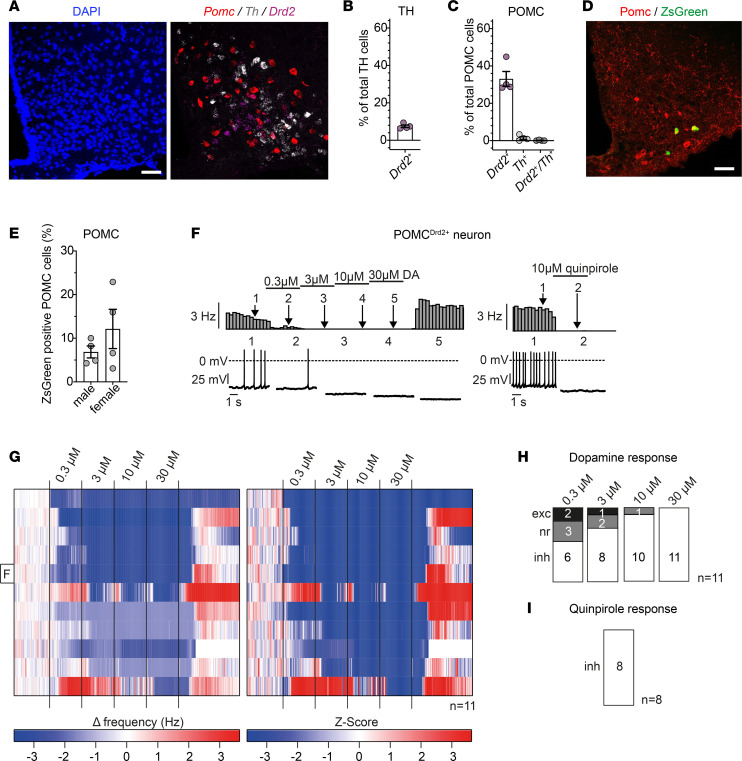
Combinatorial use of Cre and Dre recombinases allows for intersectional targeting of POMC^Drd2+^ neurons. (**A**) RNA in situ hybridization against *Pomc*, *Th*, and *Drd2* in ARC of C57BL/6N mice. Scale bar: 50 μm. (**B** and **C**) Colocalization percentages of *Drd2* with ARC TH (**B**) or POMC (**C**) neurons as quantified from RNA in situ hybridization in C57BL/6N mice (**A**). Overlap of TH neurons with POMC neurons is shown in **C**. Data are represented as mean ± SEM; *n* = 4. No statistical tests were applied. (**D**) Immunofluorescence staining against POMC and ZsGreen in ARC of POMC^Dre^ Drd2^Cre^ R26-lx-rx-ZsGreen mice. Scale bar: 50 μm. (**E**) Percentage of ZsGreen^+^ neurons over total POMC neuronal population in male and female POMC^Dre^ Drd2^Cre^ R26-lx-rx-ZsGreen mice as quantified from immunofluorescence staining (**D**). Data are represented as mean ± SEM; *n* = 4. *P* values were calculated using unpaired 2-tailed Student’s *t* tests. (**F**) Representative perforated patch-clamp recordings with rate histograms (top) and original traces (bottom) of a single POMC^Drd2+^ neuron with increasing dopamine concentrations (0.3 μM, 3 μM, 10 μM, 30 μM) and subsequent application of the Drd2 agonist quinpirole at 10 μM. Data are also shown as part of **G** at the indicated row. (**G**) Heatmap of 11 perforated patch-clamp recordings of POMC^Drd2+^ neurons in male and female POMC^Dre^ Drd2^Cre^ R26-lx-rx-ZsGreen mice. Changes in action potential frequency from baseline and corresponding *z* scores with increasing dopamine concentrations (0.3 μM, 3 μM, 10 μM, 30 μM) are depicted on the left and right, respectively. (**H** and **I**) Statistical quantification of dopamine responses (**H**) from **G** or quinpirole responses (**I**) in POMC^Drd2+^ neurons. A neuron was considered responsive if the change in firing frequency induced by drug application was 3 times larger than the SD.

We next investigated whether Drd2 may also be expressed as autoreceptors on dopaminergic TH-expressing ARC cells. Therefore, we performed RNA in situ hybridizations in ARC of C57BL/6N mice against *Pomc*, *Drd2*, and *Th*, the rate-limiting enzyme of catecholamine synthesis (Figure 4A). Here, only a minority of *Th*^+^ cells in the ARC expressed *Drd2* (7.5% ± 0.7%) (Figure 4B); however, the POMC neuronal population and the POMC^Drd2+^ neuronal subpopulation (33.1% ± 4.0% of POMC neurons) did not coexpress *Th* (1.4% ± 0.7% and 0.1% ± 0.1%, respectively) (Figure 4C). We thus reveal POMC^Drd2+^ neurons in the ARC represent a population distinct from local dopaminergic cells, clearly arguing against a role for Drd2 to act as dopaminergic autoreceptor on ARC TH neurons.

To specifically address the role of Drd2-dependent signaling in POMC^Drd2+^ neurons, we employed our Cre/Dre-dependent intersectional, dual-recombinase-based targeting strategy (4) to directly mark Drd2-expressing POMC neurons. To this end, we crossed POMC^Dre^ Drd2^Cre^ double-transgenic mice with those allowing for restricted expression of the fluorophore ZsGreen solely in the presence of both Cre and Dre recombinases (R26-lx-rx-ZsGreen) (Supplemental Figure 2, A and B). Immunofluorescence stainings of POMC^Dre^ Drd2^Cre^ R26-lx-rx-ZsGreen mice revealed that 6.8% ± 1.4% of POMC cells expressed ZsGreen in male animals, while this proportion was higher (12.1% ± 4.5%) in female mice (Figure 4, D and E). Having employed intersectional genetics to specifically label POMC^Drd2+^ neurons, we performed electrophysiological patch-clamp recordings on these cells (Figure 4, F–I). Strikingly, all tested neurons responded to dopamine incubation with a profound inhibition of firing frequency (11 out of 11 neurons at 30 μM dopamine; Figure 4H) and in response to the Drd2-selective agonist quinpirole (8 out of 8 neurons; Figure 4I).

### POMC^Drd2+^ neurons exhibit a differential translational signature compared with the global POMC neuron population.

**Figure 5:**
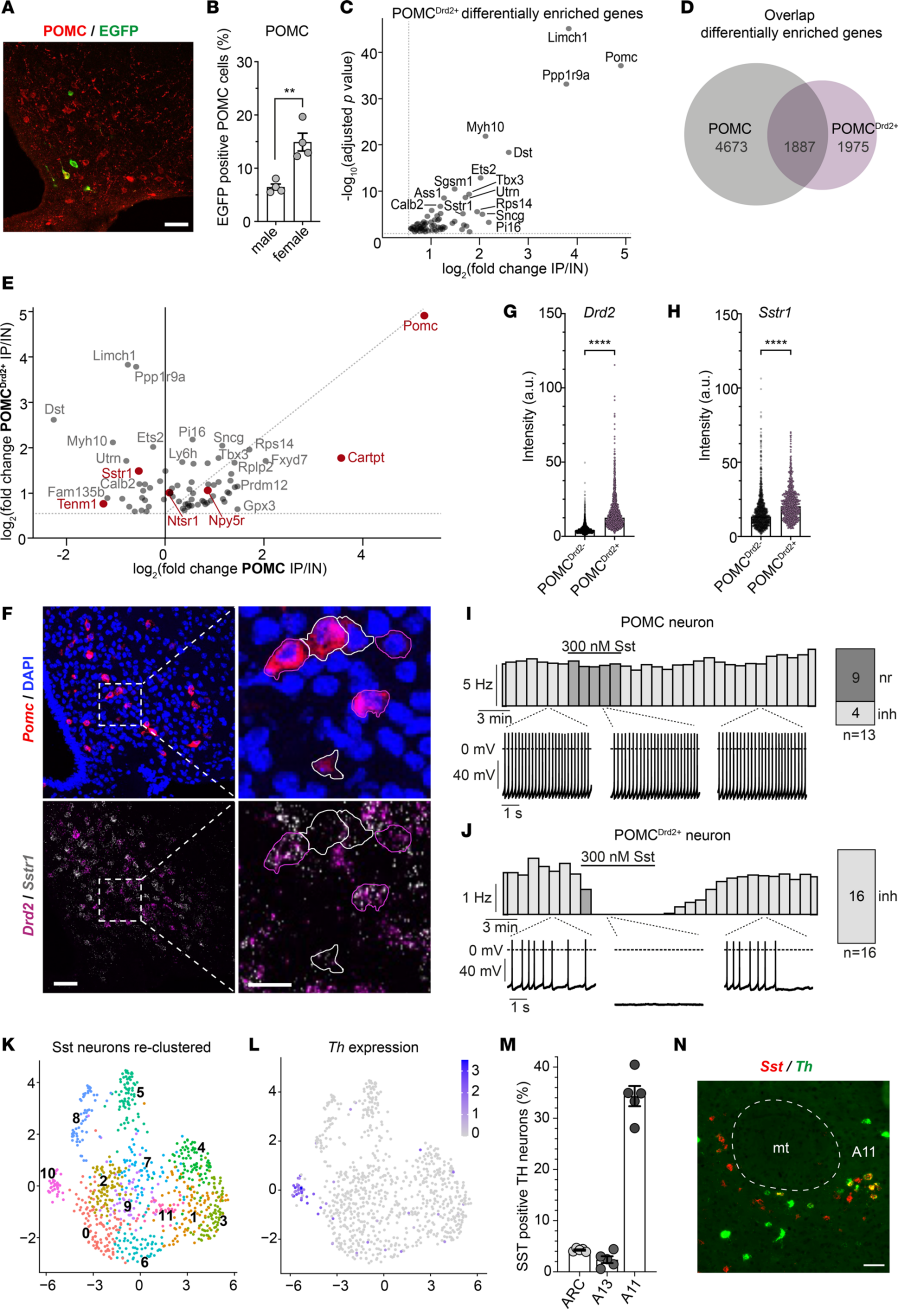
Translational profiling of POMC^Drd2+^ neurons indicates enriched Sst responsiveness compared with Drd2^–^ POMC neurons. (**A** and **B**) Immunofluorescence staining in ARC of POMC^Dre^ Drd2^Cre^ R26-lx-rx-EGFP-L10a mice. (**A**) Scale bar: 50 μm. (**B**) Percentage of GFP^+^ POMC neurons. Data are represented as mean ± SEM; *n* = 4. *P* values were calculated using unpaired 2-tailed Student’s *t* tests. **P < 0.01. (**C**) Differentially enriched genes in immunoprecipitation (IP) versus input (IN) of bacTRAP hypothalamus samples of POMC^Dre^ Drd2^Cre^ R26-lx-rx-EGFP-L10a mice. *n* = 3 replicates from *N* = 56 mice. Differential gene expression analysis was performed using the DESeq2 1.28.0 (62) R package. (**D**) Overlap of differentially enriched genes in bacterial artificial chromosome — translating ribosome affinity purification (bacTRAP) samples of POMC^Dre^ Drd2^Cre^ R26-lx-rx-EGFP-L10a and POMC^Dre^ R26-rx-EGFP-L10a mice. (**E**) Gene enrichment in POMC^Drd2+^ versus whole POMC population. Genes belonging to GO term neuropeptide signaling pathway (GO:0007218) are highlighted. For POMC^Drd2+^: *n* = 3 replicates from *N* = 56 mice. For POMC: *n* = 3 replicates from *N* = 9 mice. (**F**–**H**) RNA in situ hybridization in ARC of C57BL/6N mice. (**F**) POMC^Drd2–^ neurons are outlined in white, POMC^Drd2+^ neurons in magenta. Scale bars: 50 μm (left), 20 μm (right). (**G** and **H**) Signal intensity quantification of *Drd2* (**G**) and *Sstr1* (**H**). Data are represented as median ± SEM. *n* = 1,301–2,961 POMC^Drd2–^ and *n* = 674–1,468 POMC^Drd2+^ cells from *N* = 5 mice. *P* values were calculated using 2-tailed Mann-Whitney rank tests. ****P < 0.0001. (**I** and **J**) Perforated patch clamp recordings of Sst-treated POMC neurons (**I**) or POMC^Drd2+^ neurons (**J**). A neuron was considered responsive if the change in firing frequency induced by drug application was 3 times larger than the SD. (**K** and **L**) Reclustering of Sst neurons from Campbell et al. (2) (**K**) and corresponding *Th* RNA expression levels (**L**). (**M** and **N**) RNA in situ hybridization in C57BL/6N mice. (**M**) Percentages of *Sst* and *Th* coexpression in hypothalamic and subthalamic dopaminergic brain regions. Data are represented as mean ± SEM; *n* = 5. No statistical tests were applied. mt, mammillothalamic tract; A11, A11 dopamine cells. (**N**) Representative image of A11 dopaminergic cell group. Scale bar: 50 μm.

To further characterize the POMC^Drd2+^ neuronal subpopulation molecularly in an unbiased manner, we crossed POMC^Dre^ Drd2^Cre^ double-transgenic mice with those allowing for restricted expression of a fusion protein of the ribosomal protein L10a and EGFP solely in presence of both recombinases (R26-lx-rx-EGFP-L10a) (Supplemental Figure 3A and Figure 5, A and B). Pull-down of EGFP-tagged ribosomes from POMC^Drd2+^ neurons and subsequent mRNA sequencing of ribosome-associated mRNAs allowed for enrichment of actively translated mRNAs of these neurons. Comparing the DESeq2-normalized counts of samples after immunoprecipitation (IP) with those of the hypothalamic input (IN) samples (Figure 5C) revealed an evident, though not statistically significant, enrichment (28.6-fold ± 9.4-fold) of *Pomc* mRNA. Likewise, we found enrichment (2.5-fold ± 0.6-fold) of *Drd2* mRNA in IP samples from POMC^Drd2+^ neurons (Supplemental Figure 3B).

Analogous to the experiments described above, we performed RNA sequencing (RNA-Seq) of ribosome-associated mRNA from the global POMC neuronal population targeted by the POMC^Dre^ transgene. To this end, we crossed POMC^Dre^ mice with those expressing EGFP-L10a in a Dre-only-dependent manner (R26-rx-EGFP-L10a). Comparing the DESeq2-normalized counts of IP to IN hypothalamic samples of these mice, we found a comparable *Pomc* mRNA enrichment as observed in POMC^Drd2+^ neurons (42.2-fold ± 6.4-fold), while enrichment of *Drd2* mRNA expression was not detectable in the global POMC population (1.0-fold ± 0.3-fold) (Supplemental Figure 3C). These data support the successful enrichment of translational profiles from POMC neurons in general and the POMC^Drd2+^ subpopulation in particular.

Further, we compared the gene set size of our differential enrichment analyses of the global POMC population and the POMC^Drd2+^ subpopulation (Figure 5D). Although approximately half (48.9%) of differentially enriched genes of the POMC^Drd2+^ subpopulation were enriched in the global POMC population, the other half was exclusively enriched in POMC^Drd2+^ IP samples over their respective hypothalamic inputs. To define those mRNAs enriched in either of the investigated neuronal populations, we focused on transcripts belonging to the Gene Ontology (GO) term neuropeptide signaling pathway (GO:0007218) (Figure 5E). This analysis revealed significant enrichment (log_2_ fold change 1.5 ± 0.1) of the somatostatin receptor 1 (*Sstr1*) in POMC^Drd2+^ neurons, while its expression in the global POMC neuronal population did not exceed the hypothalamic background expression. Moreover, while other transcripts, e.g., *Npy5r*, were found to be enriched to a similar extent in POMC and POMC^Drd2+^ neurons (log_2_ fold change 0.8 ± 0.1 and 1.1 ± 0.1, respectively), we found the cocaine and amphetamine induced transcript (*Cartpt*) to be enriched to a higher extent in the global POMC population (log_2_ fold change 3.6 ± 0.2) compared with the POMC^Drd2+^ neuronal subpopulation (log_2_ fold change 1.4 ± 0.7). Taken together, despite an expectedly large overlap of enriched genes in POMC neurons in general and in the POMC^Drd2+^ neuronal subpopulation, their translational profiles diverge, which may functionally influence responses to feeding-regulatory neuropeptides.

To validate these findings, we assessed mRNA expression of *Sstr1* in POMC^Drd2+^ or POMC^Drd2–^ neurons in the ARC of C57BL/6N mice via RNA in situ hybridization (Figure 5, F–H). These analyses verified the expected enrichment of *Drd2* expression in POMC^Drd2+^ neurons (median intensity 3.8 in POMC^Drd2–^ and 12.5 in POMC^Drd2+^) but also significantly higher expression levels of *Sstr1* in this neuronal subpopulation compared with POMC^Drd2–^ cells (median intensity 13.4 in POMC^Drd2–^ and 20.3 in POMC^Drd2+^cells).

To define the potential functional consequences of this differential neuropeptide receptor expression, we compared the electrophysiological responses of POMC neurons in general and POMC^Drd2+^ neurons specifically to Sst. We therefore used ARC slices of POMC^Dre^ R26-rx-ZsGreen mice and of POMC^Dre^ Drd2^Cre^ R26-lx-rx-ZsGreen mice to perform patch-clamp recordings. Sst application to the global POMC neuronal population caused no change in firing frequency in the majority of neurons (9 out of 13 neurons) or a decrease in firing frequency in few neurons (4 out of 13 neurons) (Figure 5I). However, in a comparable cell number of the POMC^Drd2+^ subpopulation, all neurons reacted to Sst treatment with strong inhibition (Figure 5J).

Given the well-described Sst expression in orexigenic neurons of the ARC, we next employed single-cell RNA expression data, published by Campbell et al. (2), to investigate the potential coexpression of *Sst* and *Th* in the mediobasal hypothalamus. Based on the original annotation, we subsetted the data set to all cells in the 4 clusters “Agrp/Sst,” “Sst/Nts,” “Sst/Unc13c,” and “Sst/Pthlh” that express *Sst* and thus obtained 807 cells (Supplemental Figure 3, E and F). Of these cells, 76.6% expressed *Slc32a1* (Vgat) and 6.8% expressed *Th*. After reclustering the *Sst*^+^ subset at a higher resolution, we identified 1 small cluster comprising 34 cells that included the majority of all *Th*-expressing Sst neurons (85.3% *Th*^+^) (Figure 5, K and L). Additional marker genes that differentiated it from the other *Sst*^+^ clusters included polo like kinase 2 and glypican 3. To define the anatomical localization of GABAergic, *Sst*-expressing TH neurons in the hypothalamus, we performed RNA in situ hybridization with probes directed to *Slc32a1*, *Sst*, and *Th* (Figure 5, M and N, and Supplemental Figure 3, H and I). This analysis revealed low percentage of *Sst* and *Th* coexpression in the ARC (4.3% ± 0.1%) and in the A13 dopaminergic cell cluster (2.4% ± 0.7%). In contrast, TH neurons of the A11 dopaminergic cell group exhibited a higher degree of *Sst*/*Th* coexpression (34.3% ± 2.0%).

### Chemogenetic activation of POMC^Drd2+^ neurons regulates food intake and temperature preservation.

Having identified POMC^Drd2+^ neurons as a distinct subpopulation of POMC neurons, we addressed the question whether chemogenetic activation of Drd2-expressing POMC neurons could alter energy homeostasis in vivo. To this end, we crossed POMC^Dre^ Drd2^Cre^ double-transgenic mice with those allowing for restricted expression of the activatory DREADD receptor hM3Dq solely in the presence of both Cre and Dre recombinases (R26-lx-rx-hM3Dq-ZsGreen).

Given the high prevalence of Drd2-expressing POMC neuroendocrine cells in the pituitary gland, we assessed several molecular and functional parameters of pituitary gland function, to rule out impacts on whole-body metabolism by chemogenetic activation of those pituitary POMC^Drd2+^ cells. Over the course of 24 hours after clozapine *N*-oxide (CNO) administration, we observed no alterations in circulating α-melanocyte stimulating hormone (αMSH) or corticosterone concentrations between POMC^Dre^ Drd2^Cre^ R26-lx-rx-hM3Dq-ZsGreen mice and control littermates (Supplemental Figure 4, C and D). Moreover, mRNA expression levels of *Pomc* and various apoptosis and stress response markers remained unaltered in pituitary glands of the same animals (Supplemental Figure 4, E–L). These data argue against major endocrine regulatory consequences of CNO-dependent Ca^2+^-increase in Drd2-expressing POMC cells of the pituitary gland.

**Figure 6:**
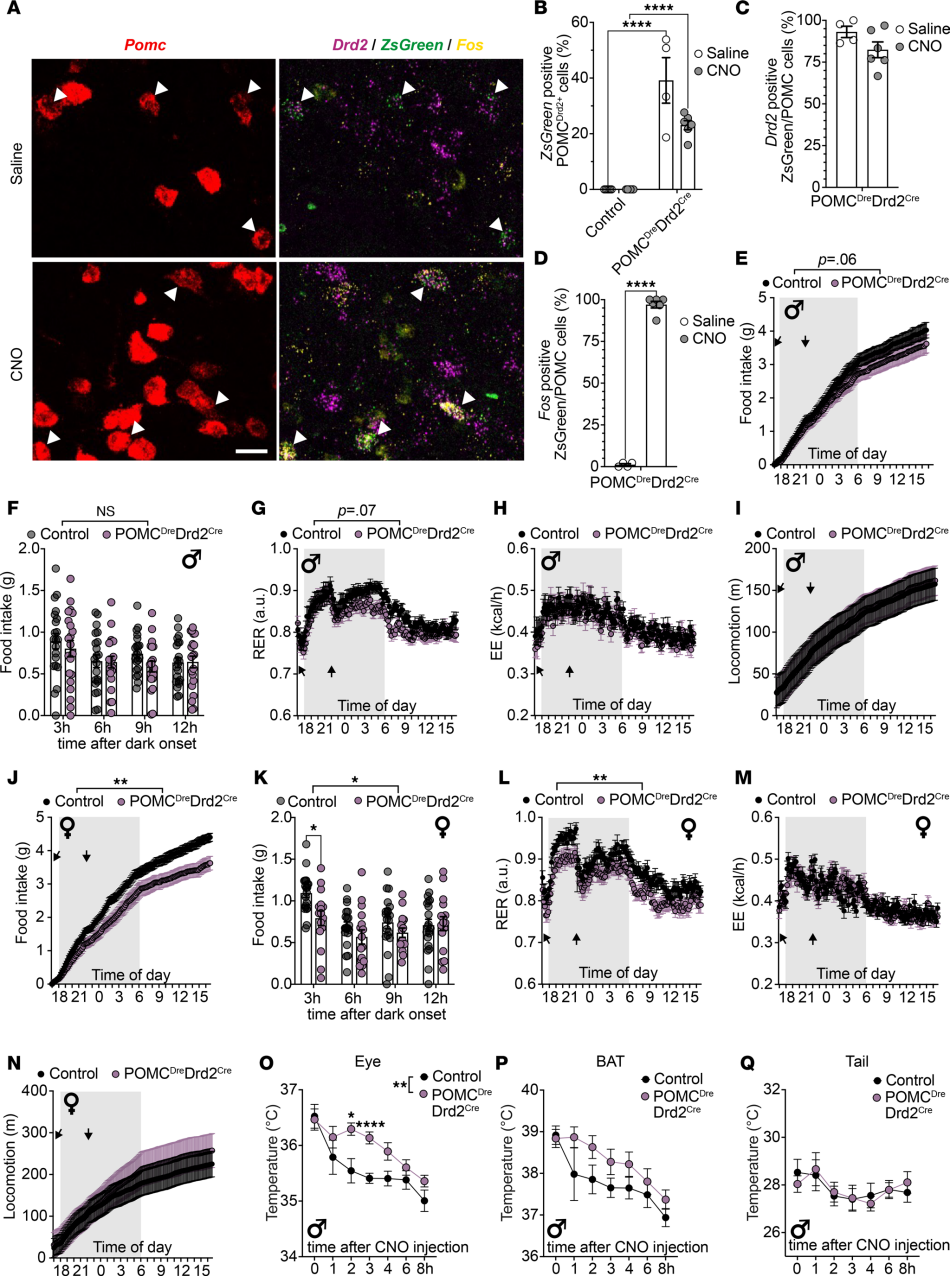
Chemogenetic activation of POMC^Drd2+^ neurons reduces food intake and increases body temperature. (**A**–**D**) RNA in situ hybridization against *Pomc*, *Drd2*, *ZsGreen*, and *Fos* in ARC of POMC^Dre^ Drd2^Cre^ R26-lx-rx-hM3Dq-ZsGreen mice 1 hour after saline or CNO injection. (**A**) Scale bar: 20 μm. Labeling efficiency (**B**), labeling specificity (**C**), and neuronal activation (**D**). Controls are *Drd2^Cre–/–^ POMC^Dre–/–^ R26-lx-rx-hM3Dq-ZsGreen^+/–^* and *Drd2^Cre+/–^ POMC^Dre–/–^ R26-lx-rx-hM3Dq-ZsGreen^+/–^* littermates. Data are represented as mean ± SEM; *n* = 4–6 per group. (**B**) *P* values were calculated using 2-way ANOVA with Holm-Šídák post hoc multiple comparisons test with a single pooled variance (genotypes). (**C** and **D**) *P* values were calculated using unpaired 2-tailed Student’s *t* tests. ****P < 0.0001. (**E**–**N**) Metabolic phenotyping of male (**E**–**I**) and female (**J**–**N**) POMC^Dre^ Drd2^Cre^ R26-lx-rx-hM3Dq-ZsGreen mice and control littermates (*Drd2^Cre–/–^ POMC^Dre–/–^ R26-lx-rx-hM3Dq-ZsGreen^+/–^*, and *Drd2^Cre+/–^ POMC^Dre–/–^ R26-lx-rx-hM3Dq-ZsGreen^+/–^* mice) upon CNO injection. Food intake (**E** and **J**), respiratory exchange ratio (**G** and **L**), locomotion (**H** and **M**), and energy expenditure (**I** and **N**) were measured over a 24-hour period. Gray areas indicate dark phase; arrows indicate CNO injections. Data from **E** and **J** are also depicted as 3-hour intervals during dark cycle (**F** and **K**). Data are represented as mean ± SEM; for female mice; *n* = 16–19 per group; for male mice *n* = 21–22 mice per group. *P* values were calculated using repeated measures (RM) 2-way ANOVA with Geisser-Greenhouse correction and Holm-Šídák post hoc multiple comparisons test. *P < 0.05. **P < 0.01. ****P < 0.0001. (**O**, **P**, and **Q**) Temperature of eye in lieu of whole-body temperature (**O**), brown adipose tissue (BAT) (**P**), and tail (**Q**) as assessed by thermal infrared imaging in CNO-injected of POMC^Dre^ Drd2^Cre^ R26-lx-rx-hM3Dq-ZsGreen mice and control littermates (*Drd2^Cre–/–^ POMC^Dre–/–^ R26-lx-rx-hM3Dq-ZsGreen*^+/–^, *Drd2^Cre+/–^ POMC^Dre–/–^ R26-lx-rx-hM3Dq-ZsGreen^+/–^*, and *Drd2^Cre–/–^ POMC^Dre+/–^ R26-lx-rx-hM3Dq-ZsGreen^+/–^* mice). Data are represented as mean ± SEM; for thermography of the eye *n* = 23, for thermography of BAT and tail *n* = 11–12 mice per group. *P* values were calculated using mixed effects model (**O**) or RM 2-way ANOVA (**P** and **Q**) with Geisser-Greenhouse correction and Holm-Šídák post hoc multiple comparisons tests.

We focused on POMC^Drd2+^ neurons in the ARC of POMC^Dre^ Drd2^Cre^ R26-lx-rx-hM3Dq-ZsGreen mice. Similar to the above-described mouse models (Figure 4E and Figure 5B), we observed a higher proportion of genetically targeted POMC^Drd2+^ neurons in females (16.3% ± 2.3%) than males (7.5% ± 0.3%) (Supplemental Figure 4M). However, RNA in situ hybridization against *Pomc*, *Drd2*, *ZsGreen*, and *Fos* as markers for neuronal activity revealed profound specificity (saline 93.1% ± 3.4%; CNO 82.4% ± 4.7% of *Drd2* in ZsGreen/POMC neurons) and robust CNO-induced neuronal activation (saline 1.1% ± 0.7%; CNO 97.0% ± 2.0% of *Fos* in ZsGreen/POMC neurons) across sexes (Figure 6, A–D).

We next performed indirect calorimetry combined with continuous monitoring of food intake in POMC^Dre^ Drd2^Cre^ R26-lx-rx-hM3Dq-ZsGreen mice. Interestingly, while male animals only showed a trend toward an anorexigenic effect and lower respiratory exchange ratio (RER) upon CNO administration compared with control littermates (Figure 6, E–G), female mice significantly and acutely responded with a decrease in food intake (16.2% ± 0.1% reduction over dark phase and additional 30.7% ± 0.1% reduction during light phase) and concomitant RER shift toward increased lipid utilization following CNO application (Figure 6, J–L). Energy expenditure and locomotion, however, remained unaffected by POMC^Drd2+^ neuron activation in both sexes (Figure 6, H, I, M, and N). Taken together, these data indicate that ARC POMC^Drd2+^ neurons are regulators of feeding and substrate metabolism.

Given the role of POMC neurons as sensors (24) and regulators (6) of body temperature, we asked whether chemogenetic activation of the Drd2-expressing POMC subpopulation may affect temperature homeostasis. We thus performed infrared thermography in CNO-injected, food-deprived POMC^Dre^ Drd2^Cre^ R26-lx-rx-hM3Dq-ZsGreen mice and control littermates. Thermal measurements of the eye as a surrogate for core temperature of control animals revealed a continuous decrease in eye temperature upon continued food deprivation. In contrast, chemogenetic activation of POMC^Drd2+^ neurons was sufficient to significantly preserve core temperature for several hours (+0.7°C ± 0.1°C 3 hours after CNO administration) (Figure 6O). Since the nonshivering responses for temperature preservation are brown adipose tissue (BAT) thermogenesis and vasoconstriction, we further assessed BAT and tail temperatures to delineate the mechanisms of temperature preservation upon chemogenetic POMC^Drd2+^ neuron activation. While comparable tail temperatures between CNO-treated POMC^Dre^ Drd2^Cre^ R26-lx-rx-hM3Dq-ZsGreen mice and control littermates indicated unaltered regulation of vasoconstriction, BAT temperature revealed a statistically nonsignificant trend toward increased BAT temperature upon chemogenetic activation of POMC^Drd2+^ neurons, which correlated with core body temperature dynamics (+0.6°C ± 0.3°C 3 hours after CNO administration) (Figure 6, P and Q).

## Discussion

Control of homeostatic and dopamine-associated feeding relies on highly interdependent pathways, and recent studies have delivered considerable evidence for direct control of neurons involved in homeostatic feeding by dopamine signaling (19, 20, 25). Here, we define and quantify dopamine-inhibited or dopamine-excited subpopulations of neurons in the melanocortin circuitry in the ARC. We find that the proportion of Drd1-expressing neurons is similar in AgRP/NPY compared with POMC neurons, which predominantly depolarize upon dopamine treatment consistent with previous studies (20). However, the notion that approximately 60% of AgRP neurons responded to dopamine with stimulation of firing frequency in the presence of synaptic blockers (Figure 2E), while only 14.4% ± 2.2% of these neurons exhibited detectable *Drd1* mRNA expression (Figure 1D), points to the possibility that even very low level of GPCR expression below the detection limit of RNA in situ hybridization may confer dopamine responsiveness. Drd2 expression, in contrast, was much higher in POMC compared with AgRP/NPY neurons, and dopamine stimulation elicited hyperpolarization in a large proportion of these cells. However, POMC neurons are not exclusively dopamine inhibited, as a certain number depolarized upon dopamine stimulation, which is in line with the ratios of those expressing Drd1 and Drd2 identified in the present study and consistent in the RNA-Seq data by Henry et al. (21) (Figure 1B). On the other hand, specifically isolating the POMC^Drd2+^ neuronal subpopulation unequivocally enriches for cells, which exhibit dopaminergic inhibition consistent with previous studies indicating Drd2-mediated POMC neuron inhibition (20). Despite the fact that previous studies had reported a predominant inhibition of POMC neurons by dopamine, the present analysis reveals the nature of the more complex dopamine-dependent regulation of this cell type, yet it unequivocally demonstrates that genetically targeted POMC^Drd2+^ neurons represent a distinct cluster of POMC neurons with profound dopamine-evoked inactivation.

Our molecular profiling of POMC^Drd2+^ neurons characterizes these cells as a dopamine-sensitive neuronal population with an enrichment for *Sstr1* expression, which translates into an electrophysiologically validated enrichment for Sst responsiveness, i.e., Sst-evoked neuronal inhibition. In fact, the proportion of Sst-inhibited POMC neurons of the global population reflects the proportion of POMC neurons expressing Drd2. Interestingly, it has been reported that Drd2 forms heteromeric complexes with Sstr5, enhancing its functional signaling activity (26). Although we have not investigated potential Sstr1/Drd2 receptor dimerization here, and any such hypothesis in the context of POMC neurons remains speculative, this finding underlines the potential synergism of dopamine and Sst signaling, by integrated signaling or through parallel inhibition of POMC^Drd2+^ neurons. Sst is known to be coexpressed in orexigenic cell types in the ARC, such as AgRP/NPY neurons, which promote feeding in response to starvation (27, 28), and Pnoc-expressing neurons in the ARC, which promote hyperphagia upon HFD feeding (29). Although clusters of AgRP/Sst and Pnoc/Sst coexpressing neurons have been identified (2, 29), and neuronal activity of both populations inhibits POMC neurons via GABA release (29, 30), it appears as a common mechanism that co-release of Sst from feeding-stimulatory neurons can also engage Sst signaling to inhibit food intake–suppressing POMC neurons.

Along this line, our analysis revealed the coexpression of *Th* and *Sst*, which was enriched in the dopaminergic A11 cell group. We herein support the previously described GABAergic nature of TH^ARC^ neurons (31, 32) and further hypothesize that a subset of ARC, A13, and most prominently A11 TH neurons may co-release GABA, Sst, and dopamine to inhibit POMC^Drd2+^ neurons. This is further supported by the notion that ARC neurons receive synaptic input from dopaminergic neurons in the zona incerta, capable of modulating feeding (33). Thus, future studies clearly have to investigate the potential mechanisms of multitransmitter integration in the coordinate regulation of POMC neuron activity in control of feeding.

In addition to coordinated synaptic co-release of GABA, Sst, and dopamine, an alternative model of dopamine-evoked regulation of ARC melanocortin neurons may include the mechanism of volume transmission. Zhang and Van Den Pol hypothesize that despite undetectable direct synaptic contacts of AgRP/NPY and TH neurons in the ARC, AgRP/NPY neurons may still receive their dopaminergic input by TH^ARC^ cells via volume transmission (34).

Furthermore, in dopamine signaling in the hypothalamus, it is crucial to consider the impact of neuroendocrine dopamine (NEDA) neurons, which release dopamine to regulate pituitary gland functions. NEDA neurons can be classified into 3 groups according to anatomical location and projection sites: first, tuberoinfundibular dopaminergic (TIDA) neurons in the ARC, which release dopamine into the portal capillary vessel of the median eminence to ultimately act on cells in the anterior lobe (AL) of the pituitary gland (35); second, tuberohypophyseal dopaminergic neurons in the rostral ARC; and third, periventricular-hypophysial dopaminergic neurons in the periventricular nucleus of the hypothalamus, both of which directly innervate the intermediate lobe (IL) of the pituitary gland (36, 37). Pituitary cells in the AL under dopaminergic regulation include prolactin-releasing (PRL-releasing) lactotrophs and adrenocorticotropic hormone–releasing corticotrophs (38, 39). PRL has long been known for its orexigenic effects (reviewed in ref. 40); however, Drd2 agonism in humans was shown to trigger BW loss and increase in resting energy expenditure in a PRL-independent manner (41). Since TIDA neurons in the ARC release dopamine into the interstitial space at the median eminence to ultimately inhibit PRL release from lactotrophs, TIDA neurons likely also provide an unspecific source of dopamine input both for POMC and AgRP/NPY neurons (as discussed above). Corticotrophs in the AL and the majority of cells in the IL of the pituitary gland represent Drd2-expressing POMC cells (39, 42). Our intersectional targeting approach reveals transgenic labeling of all POMC cells in the IL, yet no labeling of POMC-expressing cells in the AL was detected. Chronic activation of IL melanocytes in a chronic stress model leads to ER stress and cellular degradation in rats (43). Conversely, Drd2 antagonist treatment, i.e., disinhibition of melanocytes in the IL, leads to increased POMC expression and cell number of POMC neuroendocrine cells in the IL (44). Therefore, in our in vivo model of chemogenetic activation of POMC^Drd2+^ neurons, we assessed serum corticosterone and αMSH levels, and our data argued against major endocrine regulatory consequences of Ca^2+^ increase in Drd2-expressing POMC cells of the pituitary gland. Furthermore, we found no indication for increased cell death, ER stress, or altered POMC expression in our model.

Our data show that POMC^Drd2+^ neurons of the ARC are functionally capable of suppressing feeding and regulating core body temperature. We identify a vast populational overlap between POMC^Drd2+^ and POMC^Glp1r+^ neurons, in agreement with the food intake–regulatory effect of POMC^Glp1r+^ neurons (4). In fact, our group demonstrated activation of the proportionally larger targeted subpopulation of Lepr-expressing POMC neurons (~45% POMC^Lepr+^ and 37% POMC^Glp1r+^ of all POMC neurons) did not elicit food intake inhibition to the same extent as POMC^Glp1r+^ or POMC^Drd2+^ neurons. However, as the observed sex bias in our intersectional targeting strategy for POMC^Drd2+^ neurons (16.3% in female vs. 7.5% in male POMC^Drd2+^ of all POMC neurons) caused a significant 16.2% food reduction over the duration of the dark phase in female mice, but a milder and nonsignificant (*P* = 0.06) reduction in male animals, we hypothesize the sex differences in food intake regulation scale with the number of transgenically targeted neurons. Consistent with the higher targeted POMC^Drd2+^ neurons in females compared with male littermates, we observed a slightly increased proportion of endogenously Drd2-expressing POMC neurons in female compared with male control mice (Supplemental Figure 4N). Combined with the predescribed higher expression of POMC in POMC neurons of female compared with male mice (22), these 2 factors may have contributed to the differential recombination efficiency. Moreover, our electrophysiological experiments indicated a higher sensitivity for dopamine-elicited inhibition of POMC neurons in female compared with male mice. Overall, these data argue the hereto-observed sex differences may not be exclusively explained by technical limitations but may reflect a physiological sex difference in dopamine responses. In this context the limitation of our experimental model has to be pointed out, that its high selectivity of targeting neuronal subpopulations comes at the expense of a relatively low efficiency to target those neurons. Nevertheless, we could show that our intersectional targeting approach suits the purpose of clearly highlighting the important role for POMC^Drd2+^ in feeding. In fact, Folgueira et al. showed that chemogenetic activation of all Drd2-expressing neurons in the mediobasal hypothalamus had no effect on feeding or interscapular temperature in mice (41). While this study design may also target POMC-antagonistic signaling circuitries in the hypothalamus, including Drd2-expressing AgRP neurons, our experimental model provides the necessary cellular specificity to attribute feeding and temperature regulation to POMC^Drd2+^ neurons in the hypothalamus.

Although the sympathetic nervous system–driven activation of BAT thermogenesis through POMC signaling has been widely acknowledged, the proposed thermoregulatory function of POMC neurons is substantially based on studies downstream of the engagement of the melanocortin-4 receptor, by POMC-derived peptides, namely αMSH (45, 46). However, the direct impact of POMC neurons on thermogenesis, particularly in a feeding-independent manner, remained to be elucidated. Here, we show POMC neurons are at least in part capable of controlling BAT temperature directly, by revealing the activation of the POMC^Drd2+^ neuronal subpopulation is sufficient to modulate core body temperatures independent of its feeding-regulatory function in food-deprived mice.

Taken together our data define POMC^Drd2+^ neurons as a dopamine-inhibited, molecularly defined subpopulation of POMC neurons with enhanced Sst responsiveness. Finally, we reveal a feeding- and thermoregulatory function for POMC^Drd2+^ neurons. We thus demonstrate the molecular and functional significance of dopamine signaling in neurons of the melanocortin circuitry in energy homeostasis.

## Methods

### Resource availability

#### Lead contact.

Further information and requests for resources and reagents should be directed to and will be fulfilled by the lead contact, Jens C. Brüning (bruening@sf.mpg.de).

#### Data and code availability.

RNA-Seq data of ribosome-associated mRNA (bacTRAP) data of POMC and POMC^Drd2+^ neurons have been deposited at NCBI Gene Expression Omnibus (GEO) and are publicly available as of the date of publication (accession GSE210311).

Any additional information required to reanalyze the data reported in this paper is available from the lead contact upon request.

### Experimental model and subject details

#### Mouse husbandry.

All animal procedures were conducted in compliance with protocols approved by the local authorities (Bezirksregierung Köln). Permission for breeding and experiments on mice was issued by the Department for Environment and Consumer Protection - Veterinary Section, Cologne, North Rhine-Westphalia, Germany [(§11) 576.1.35.2.G 07/18, 84-02.04.2017.A058]. Mice were group-housed in individually ventilated cages at 22°C–24°C with 12-hour light/12-hour dark cycle and ad libitum access to water and normal chow diet (NCD; ssniff Spezialdiäten, catalog V1554-703) containing 67 kJ% carbohydrate, 23 kJ% protein, and 10 kJ% fat. Mice had restricted access to food only for time-limited periods during glucose and body temperature measurements via infrared thermography (Teledyne FLIR, FLIR E6-XT) and immediately before organ harvest. Group-housing was randomized by weaning pups into allocated cages of 2 to 5 animals without prior knowledge of genotypes. Mice were single-housed to measure indirect calorimetry (Promethion, Sable Systems) or to determine body temperature via infrared thermography. Following these procedures, female mice were regrouped into their original cage distributions, while male mice remained single-housed.

#### Mouse lines.

C57BL/6N mouse line was obtained from Charles River. For RNA in situ hybridization 12-week-old male mice were used.

NPY^GFP^ [B6.FVB-Tg(Npy-hrGFP)1Lowl/J] mouse line has been described (47) and was obtained from Jackson Laboratory (stock number: 006417; RRID: IMSR_JAX:006417). *NPY^GFP+/–^* mice of both sexes were used for electrophysiological studies at age between 11 and 20 weeks.

POMC^GFP^ [C57BL/6J-Tg(Pomc-EGFP)1Low/J] mouse line has been described (48) and was obtained from Jackson Laboratory (stock number: 009593; RRID: IMSR_JAX:009593). *POMC^GFP+/–^* mice of both sexes were used for electrophysiological studies at age 11–20 weeks.

Drd2^Cre^ [Tg(Drd2-cre)ER44Gsat] mouse line has been described (49) (MGI: 3836635).

POMC^Dre^ mouse line has been generated in our laboratory and been described (4).

R26-lx-rx-ZsGreen (ROSA26-CAGS-lox-STOP-lox-rox-STOP-rox-ZsGreen) mouse line has been described (50).

R26-lx-rx-EGFP-L10a and R26-lx-rx-hM3Dq-ZsGreen mouse lines have been generated in our laboratory and been described (4). Mice have originally been named “ROSA26lSlrSrEGFPL10a” and “ROSA26lSlrSrhM3Dq.”

R26-rx-EGFP-L10a and R26-rx-ZsGreen mouse lines have been generated in our laboratory by crossing previously published (4, 50) R26-lx-rx-transgenic mice to mice that ubiquitously express Cre recombinase [BALB/c-Tg(CMV-cre)1Cgn/J, Jackson Laboratory stock number: 003465; RRID: IMSR_ JAX:003465] (51), which were kept in-house on a C57BL/6N background.

All above-listed mouse lines were maintained on and regularly backcrossed to a C57BL/6N background (Charles River) in the facility of the Max Planck Institute for Metabolism Research. For breeding purposes of animals, refer to *Breeding strategies*.

#### Breeding strategies.

Both for male and female mice, 8 weeks was considered the minimum age for all crossings.

NPY^GFP^, POMC^GFP^, Drd2^Cre^, and POMC^Dre^ mice were heterozygously maintained by crossing *NPY^GFP+/–^*, *POMC^GFP+/–^*, *Drd2^Cre+/–^*, or *POMC^Dre+/–^* to WT C57BL/6N animals (Charles River). *NPY^GFP+/–^* or *POMC^GFP+/–^* animals were consequently used for electrophysiological recordings, *Drd2*^Cre+/–^ and *POMC^Dre+/–^* mice were crossed to each other to obtain *Drd2^Cre+/–^ POMC^Dre+/–^* double-transgenic mice, which were subsequently bred with the R26-lx-rx-transgenic animals.

R26-lx-rx-ZsGreen, R26-lx-rx-EGFP-L10a, and R26-lx-rx-hM3Dq-ZsGreen were homozygously maintained. *R26-lx-rx-transgenic^+/+^* mice were crossed to *Drd2^Cre+/–^ POMC^Dre+/–^* double-transgenic animals to obtain experimental animals of 4 possible genotypes: *Drd2^Cre–/–^ POMC^Dre–/–^ R26-lx-rx-transgenic^+/–^*, *Drd2^Cre–/–^ POMC^Dre+/–^ R26-lx-rx-transgenic^+/–^*, *Drd2^Cre+/–^ POMC^Dre–/–^ R26-lx-rx-transgenic^+/–^*, or *Drd2^Cre+/–^ POMC^Dre+/–^ R26-lx-rx-transgenic^+/–^* mice. The latter (triple-transgenic *Drd2^Cre+/–^ POMC^Dre+/–^ R26-lx-rx-transgenic^+/–^* mice) are referred to as “POMC^Dre^ Drd2^Cre^ R26-lx-rx-transgenic” animals throughout the manuscript. For genotype controls *Drd2^Cre–/–^ POMC^Dre–/–^ R26-lx-rx-transgenic^+/–^*, *Drd2^Cre–/–^ POMC^Dre+/–^ R26-lx-rx-transgenic^+/–^*, and *Drd2^Cre+/–^ POMC^Dre–/–^ R26-lx-rx-transgenic^+/–^* have been used as indicated in the respective method description. Metabolic phenotyping of *Drd2^Cre–/–^ POMC^Dre+/–^R26-lx-rx-transgenic^+/–^* mice has been described in detail (4) and is not further addressed in this manuscript. Resulting POMC^Dre^ Drd2^Cre^ R26-lx-rx-ZsGreen animals and control littermates were used for immunohistological analyses and electrophysiological recordings. Resulting POMC^Dre^ Drd2^Cre^ R26-lx-rx-EGFP-L10a animals were used for immunohistological analyses and bacTRAP translational profiling. Resulting POMC^Dre^ Drd2^Cre^ R26-lx-rx-hM3Dq-ZsGreen and control littermates were used for RNA in situ hybridization analyses and metabolic phenotyping.

R26-rx-EGFP-L10a and R26-rx-ZsGreen mice were homozygously maintained. *R26-rx-transgenic^+/+^* mice were crossed to *POMC^Dre+/–^* transgenic animals to obtain experimental animals of 2 genotypes: *POMC^Dre–/–^ R26-rx-transgenic^+/–^* or *POMC^Dre+/–^ R26-rx-transgenic^+/–^* mice. The latter are referred to as “POMC^Dre^ R26-rx-EGFP-L10a” or “POMC^Dre^ R26-rx-ZsGreen” animals throughout the manuscript. Resulting POMC^Dre^ R26-rx-EGFP-L10a animals were used for bacTRAP translational profiling of the whole POMC neuronal population. POMC^Dre^ R26-rx-ZsGreen mice were used for electrophysiological recordings of the whole POMC neuronal population.

### Method details

#### RNA in situ hybridization (RNAscope).

RNA in situ hybridization was performed on tissue samples of male WT C57BL/6N mice (Charles River) at 12 weeks of age and of male and female POMC^Dre^ Drd2^Cre^ R26-lx-rx-hM3Dq-ZsGreen mice and control littermates (*Drd2^Cre–/–^ POMC^Dre–/–^ R26-lx-rx-hM3Dq-ZsGreen^+/–^* and *Drd2^Cre+/–^ POMC^Dre–/–^ R26-lx-rx-hM3Dq-ZsGreen^+/–^* mice) between 14 and 17 weeks of age, which were fasted at time point –120 minutes during the light cycle, i.p. injected with 3 mg/kg CNO in 0.9% saline at time point –60 minutes, and transcardially perfused at time point 0 minutes. Perfusion and tissue sectioning was performed as described above. To stain for RNA in situ, the RNAscope method by Advanced Cell Diagnostics was applied. All utilized reagents and probes can be found in Supplemental Table 1. In brief, slides were incubated at 60°C for approximately 6 hours, treated with 1× Target Retrieval Reagent (Advanced Cell Diagnostics, catalog 323100) at 99°C for 10 minutes, washed in sterile H_2_O for 15 seconds, washed in 100% EtOH for 3 minutes, and consequently dried overnight. The following day tissues were bordered with a hydrophobic ImmEdge Pen (Biozol, catalog VEC-H-4000) and incubated with Protease Plus (Advanced Cell Diagnostics, catalog 323100) for 25 minutes at 40°C. Samples were washed twice in sterile H_2_O for 2 minutes, and prewarmed (40°C) probe mix was applied to the sections. The probe mix contained up to 4 probes, which were amplified using the RNAscope 4-Plex Ancillary Kit for Multiplex Fluorescent Kit v2 (Advanced Cell Diagnostics, catalog 323120). All probes were applied at concentrations recommended by the manufacturer with following exceptions: Mm-Pomc was diluted 1:4, Mm-Agrp 1:2, Mm-Drd1a 1:1.5, Mm-Drd2 1:1.5, and Mm-Fos 1:2 respective to the recommended concentrations. RNAscope 4-plex negative (Advanced Cell Diagnostics, catalog 321831) and positive-control probes (Advanced Cell Diagnostics, catalog 321811) were processed in parallel with the target probes. Slides were incubated with the probe mix for 2 hours at 40°C and washed twice in 1× wash buffer (Advanced Cell Diagnostics, catalog 323100) for 2 minutes. All following incubation steps were performed at 40°C followed by a wash step as before: AMP1 for 30 minutes, AMP2 for 30 minutes, AMP3 for 15 minutes, HRP-C1 for 15 minutes, C1-fluorophore for 30 minutes, HRP blocker for 15 minutes, HRP-C2 for 15 minutes, C2-fluorophore for 30 minutes, HRP blocker for 15 minutes, HRP-C3 for 15 minutes, C3-fluorophore for 30 minutes, HRP blocker for 15 minutes, HRP-C4 for 15 minutes, and C4-fluorophore for 30 minutes. For fluorescent probe detection, the fluorophores Opal 520 (PerkinElmer, catalog FP1487001KT), Opal 570 (PerkinElmer, catalog FP1488001KT), Opal 620 (PerkinElmer, catalog FP1495001KT), and Opal 690 (PerkinElmer, catalog FP1497001KT) were applied at dilutions between 1:750 and 1:2,000 depending on the further shelf life of the reagent. Sections were incubated for 1 minute at room temperature with DAPI nuclear counterstain (Advanced Cell Diagnostics, catalog 323100), coverslipped in ProLong Gold Antifade Mountant (Thermo Fisher, catalog P36931), and stored in the dark at 4°C until imaged. All RNA in situ hybridizations were visualized on a confocal Leica TCS SP-8-X microscope.

#### Data analysis of RNA in situ hybridization (RNAscope).

Microscopic images of RNA in situ hybridization were visualized using the image-processing software ImageJ (1.53f51, NIH). For counting of cells coexpressing a certain target, the Cell Counter plug-in (by Kurt De Vos, University of Sheffield, Sheffield, United Kingdom) was used. *Agrp^+^* or *Pomc^+^* areas defined the cell region of interest (ROI) in which coexpression of the given target was evaluated; 3 or more dots of target signal within a given cell defined it as positive and less signal as negative. For signal intensity analysis, ROIs were defined outlining the *Pomc* signal: in the image channel depicting *Pomc* signal, a 2.0 pixel median filter was applied, followed by the Triangle autothresholding method for white objects on black background. Thus defined particles were filtered for a minimum size of 20 μm and defined as ROIs including holes. Correct outlining of all POMC neurons was ensured by visual judgment. Cells that were considered wrongly outlined were excluded from the analysis. POMC neurons were furthermore divided into *Drd2*^+^ or *Drd2*^–^ subpopulations by defining any cell with 3 or more dots of *Drd2* signal as positive, with less signal as negative. After ROI definition of POMC neurons and their allocation into subpopulations, they were measured for integrated density and area within the given target image channel. Intensity was defined as intensity (a.u.) = raw integrated density/area. For signal intensity analysis of RNA in situ hybridization, intensities of single cells were plotted as assigned to their respective subpopulation.

#### Electrophysiological measurements.

For electrophysiological measurements NPY^GFP^, POMC^GFP^, POMC^Dre^ Drd2^Cre^ R26-lx-rx-ZsGreen, and POMC^Dre^ R26-rx-ZsGreen mice of both sexes were used at 11–20 weeks of age. The electrophysiological experiments were carried out essentially as described (4). In brief, the animals were lightly anesthetized with isoflurane (AbbVie; catalog B506) and decapitated, and coronal brain slices of 280 μm thickness containing the ARC were cut with a vibration microtome (Leica Biosystems; Leica VT1200) under cold (4 °C), carbogenated (95% O_2_ and 5% CO_2_), glycerol-based modified artificial cerebrospinal fluid (GaCSF) (52). GaCSF contained 244 mM glycerol, 2.5 mM KCl, 2 mM MgCl_2_, 2 mM CaCl_2_, 1.2 mM NaH_2_PO_4_, 10 mM HEPES, 21 mM NaHCO_3_, and 5 mM glucose, adjusted to pH 7.2 with NaOH. Afterward, slices were transferred into carbogenated aCSF at 36°C for 30–40 minutes and kept at room temperature until further usage for electrophysiological recordings. aCSF contained 125 mM NaCl, 2.5 mM KCl, 2 mM MgCl_2_, 2 mM CaCl_2_, 1.2 mM NaH_2_PO_4_, 21 mM NaHCO_3_, 10 mM HEPES, and 5 mM glucose, adjusted to pH 7.2 with NaOH. During electrophysiological recordings, brain slices were continuously superfused with carbogenated (95% 0_2_; 5% CO_2_) aCSF at a flow rate of ~2.5 mL/min. In all recordings, the aCSF contained 10^−4^ M picrotoxin (Sigma-Aldrich; catalog P1675), 5 × 10^–6^ M CGP-54626 (Biotrend, catalog BN0597), 5 × 10^−5^ M DL-AP5 (Biotrend, catalog BN0086), and 10^−5^ M CNQX (Sigma-Aldrich, catalog C127) to block GABAergic and glutamatergic synaptic input.

Current-clamp recordings were performed at approximately 32°C in the perforated patch-clamp configuration. Neurons were visualized with a fixed-stage upright microscope (BX51WI, Olympus) using ×60 water-immersion objectives (LUMplan FL/N ×40, 0.8 numerical aperture, 2 mm working distance; LUMplan FL/N ×60, 1.0 numerical aperture, 2 mm working distance, Olympus) with fluorescence optics and infrared differential interference contrast optics (53). Neurons were identified by their anatomical location in the ARC and by their GFP or ZsGreen fluorescence that was visualized with an X-Cite 120 illumination system (EXFO Photonic Solutions) in combination with a Chroma 41001 filter set (excitation: HQ480/×40; beam splitter: Q505LP; emission: HQ535/50m). Electrodes with tip resistances between 4 and 7 MΩ were fashioned from borosilicate glass (0.86 mm inner diameter; 1.5 mm outer diameter; GB150-8P, Science Products) with a vertical pipette puller (PP-830, Narishige). All recordings were performed with an EPC10 patch-clamp amplifier (HEKA) controlled by the program PatchMaster (version 2x90; HEKA) running under Windows. In parallel, data were recorded using a micro1410 data acquisition interface and Spike 2 (version 7.01, both from CED). Current-clamp recordings were sampled at 25 kHz and low-pass-filtered at 2 kHz with a 4-pole Bessel filter. The calculated liquid junction potential of 14.6 mV between intracellular and extracellular solution was compensated (calculated with Patcher’s Power Tools plug-in from https://www3.mpibpc.mpg.de/groups/neher/index.php?page=software for Igor Pro 6; Wavemetrics).

Perforated patch experiments were conducted using protocols modified from previous studies (54, 55). Recordings were performed with a pipette solution containing 140 mM K-gluconate, 10 mM KCl, 10 mM HEPES, 0.1 mM EGTA and 2 mM MgCl_2_, adjusted to pH 7.2 with KOH. ATP and GTP were omitted from the intracellular solution to prevent uncontrolled permeabilization of the cell membrane (56). The patch pipette tip was filled with internal solution and backfilled with internal solution, which contained the ionophore amphotericin B (Sigma-Aldrich; catalog A4888) to achieve perforated patch recordings (57, 58), 0.02% tetramethylrhodamine-dextran (Invitrogen, catalog D3308) to monitor the stability of the perforated membrane, and 1 % biocytin (Sigma-Aldrich; catalog B4261) to label the recorded neuron. Amphotericin B was dissolved in DMSO to a concentration of 40 μg/μL (Sigma-Aldrich; catalog D8418) following the protocols of a previous study (4). The used DMSO concentration (0.1–0.3%) had no noticeable effect on the investigated neurons. The ionophore was added to the modified pipette solution shortly before use. The final concentration of amphotericin B was approximately 120 to 160 μg/mL. Amphotericin solutions were prepared from undissolved weighted samples (stored at 4°C protected from light) every recording day. During the perforation process, access resistance (*R*_a_) was monitored continuously, and experiments started after *R*_a_ values reached a steady state (~10–20 minutes) and the action potential amplitude was stable. To confirm the integrity of the perforated patch, *R*_a_ was monitored. A change to the whole-cell configuration was also indicated by dextran fluorescence in the cell body.

To investigate the dopamine responses in AgRP/NPY neurons of NPY^GFP^ mice or in POMC neurons of POMC^GFP^ mice, increasing dopamine (Sigma-Aldrich; catalog H8502) concentrations of 0.3 μM, 3 μM, 10 μM, and 30 μM were sequentially bath-applied for 10 minutes each concentration. To study the effect of Sst (Sigma-Aldrich; catalog S1763), in POMC neurons of POMC^Dre^ R26-rx-ZsGreen mice or in POMC^Drd2+^ neurons of POMC^Dre^ Drd2^Cre^ R26-lx-rx-ZsGreen mice, Sst was bath-applied at a concentration of 300 nM for 5 minutes.

Data analysis was performed with Spike2 (Cambridge Electronics), GraphPad Prism (version 8.2; GraphPad Software Inc), and custom-made analysis scripts written in Igor Pro. To visualize the significance of the dopamine responses, we used *z* score plots. For the bar graphs in Figures 2–4 and Supplemental Figure 1, we used the “3 times SD” (3σ) criterion to classify a neuron as a responder on the single-cell level: a neuron was considered responsive if the change in firing frequency induced by drug application was 3 times larger than the SD. Means and respective SDs of spontaneous action potential firing were calculated from 5-minute periods (divided into 30 bins, each 10 seconds long) during baseline conditions and at the end of the drug application. In neurons that did not elicit action potentials, we used changes in membrane potential in a similar way as a response indicator.

#### bacTRAP.

bacTRAP was performed on hypothalami of male and female POMC^Dre^ Drd2^Cre^ R26-lx-rx-EGFP-L10a mice or male and female POMC^Dre^ R26-rx-EGFP-L10a mice. Animals were decapitated randomly fed at 11 to 13 weeks of age, brains were quickly removed, and hypothalami were dissected using a stainless steel brain matrix (World Precision Instruments). Samples were snap-frozen in liquid nitrogen and stored at –80°C until further processing. For POMC^Dre^ Drd2^Cre^ R26-lx-rx-EGFP-L10a mice, hypothalami of 18–20 animals with balanced sex proportions were pooled per replicate for a total of 3 replicates. For POMC^Dre^ R26-rx-EGFP-L10a mice, hypothalami of 3 animals (2 males, 1 female) were pooled per replicate for a total of 3 replicates. The method for purifying translating ribosomes was performed as described by Heiman et al. (59) with minor modifications: in brief, 375 μL Protein A Dynabeads (Invitrogen, catalog 10001) per replicate were washed 3 times in wash buffer I (20 mM HEPES/pH 7.4, 5 mM MgCl_2_, 150 mM KCl, 1% Nonidet P-40, 0.5 mM DTT, and 100 μg/mL cycloheximide) and subsequently resuspended in 275 μL wash buffer I. An anti-GFP antibody mixture (50 μg of Heintz Lab TRAP anti-GFP 19C8 antibody, catalog Htz-GFP-19C8; RRID: AB_2716737 and 50 μg of Heintz Lab TRAP anti-GFP 19F7 antibody, catalog Htz-GFP-19F7; RRID: AB_2716736) was incubated with the beads overnight at 4°C with slow end-over-end mixing. The following day beads were washed 3 times in wash buffer II (20 mM HEPES/pH 7.4, 5 mM MgCl_2_, 150 mM KCl, 1% Nonidet P-40, 0.5 mM DTT, and 200 μg/mL cycloheximide) and subsequently resuspended in 200 μL wash buffer II. For lysis buffer preparation 1 tablet of cOmplete mini EDTA-free protease inhibitor cocktail (Sigma-Aldrich, catalog 11836170001) was dissolved in 10 mL 20 mM HEPES/pH 7.4, 5 mM MgCl_2_, 150 mM KCl, 0.5 mM DTT, 40 U/mL RNasin, and 100 μg/mL cycloheximide. Pooled hypothalami were homogenized in 1 mL lysis buffer on a rotating glass/teflon potter homogenizer (Braun Biotech, Potter S) at 4°C twice at 250 rpm and 9 times at 750 rpm. Homogenates were centrifuged in low binding microfuge tubes (Applied Biosystems, catalog AM12450) at 2,000*g* and 4°C for 10 minutes. Supernatants were consequently mixed on ice with Nonidet P-40 (AppliChem, catalog A1694,0250) and 1,2-diheptanoyl-sn-glycero3-phosphocholine (Avanti Polar Lipids, catalog 850306P) at a final concentration of 1% (w/v) and 30 mM, respectively; incubated for 5 minutes; and centrifuged for 10 minutes at 17,000*g* and 4°C. A total of 30 μL of the supernatant was snap-frozen in liquid nitrogen until RNA extraction and served as hypothalamic input sample for each respective IP in the analysis of translational profiling. The remaining supernatant was mixed on ice with 200 μL anti-GFP antibody-coated beads and incubated for 1 hour at 4°C with slow end-over-end mixing. Sample-bead complexes (IPs) were collected via magnet and washed 4 times with wash buffer III (20 mM HEPES/pH 7.4, 5 mM MgCl_2_, 350 mM KCl, 1% Nonidet P-40, 0.5 mM DTT, 100 μg/mL cycloheximide). RNA of hypothalamic input and IP samples was extracted using the RNeasy Micro Kit (QIAGEN, catalog 74004). In brief, samples were eluted off the beads by adding 350 μL RLT buffer and incubating for 5 minutes at room temperature. Subsequently manufacturer’s instructions were followed without alteration. RNA integrity was assessed using a 2100 Bioanalyzer (Agilent).

#### RNA-Seq.

RNA-Seq was performed on bacTRAP samples of POMC^Dre^ Drd2^Cre^ R26-lx-rx-EGFP-L10a or POMC^Dre^ R26-rx-EGFP-L10a mice. Preamplification was performed via Ovation RNA-Seq system (V2), using total RNA with both poly(T) and random primers for first-strand cDNA synthesis, followed by second-strand cDNA synthesis and isothermal strand displacement amplification. cDNA libraries were prepared from 1 ng cDNA input according to the Illumina Nextera XT DNA sample preparation protocol. After validation (Agilent 2200 TapeStation) and quantification (Invitrogen Qubit), transcriptome libraries of matching samples were pooled. Pools were quantified via Peqlab KAPA Library Quantification Kit and the Applied Biosystems 7900HT Sequence Detection and sequenced on an Illumina HiSeq 4000 instrument with a 2 × 75 bp paired-end read length protocol.

#### bacTRAP RNA-Seq analysis.

For RNA-Seq analysis of bacTRAP samples of POMC^Dre^ Drd2^Cre^ R26-lx-rx-EGFP-L10a or POMC^Dre^ R26-rx-EGFP-L10a mice, we applied the community-curated nf-core/rnaseq analysis pipeline version 3.0 (60). The gene-level quantification was carried out using Salmon 1.4.0 (61) using the reference genome GRCm38. The differential gene expression analysis was performed using the DESeq2 1.28.0 (62) R package. Protein-coding genes were filtered for using the Ensembl (63) biomaRt (64) package.

Differentially expressed genes were identified by comparing POMC^Drd2+^-IP versus POMC^Drd2+^-IN (*P*_adj_ ≤ 0.05) or POMC-IP versus POMC-IN. We furthermore filtered for cells enriched in POMC neuronal subclusters from a single-cell sequencing map of the hypothalamus (65). We additionally marked genes part of the neuropeptide signaling pathway GO term (66).

#### Reclustering single-cell RNA-Seq of Sst clusters.

For the analysis of Th and Sst coexpressing neurons, we used the processed data from Campbell et al. (2) deposited at GEO (accession GSE93374). Based on the original annotation, we subsetted the data set to all cells in the 4 clusters “Agrp/Sst”, “Sst/Nts”, “Sst/Unc13c” and “Sst/Pthlh” that express Sst to obtain 807 cells. We then reprocessed this subset using the standard Seurat pipeline (see https://satijalab.org/seurat/articles/pbmc3k_tutorial.html) (67) with a small number of highly variable features, 400, and principal components, 20. For reclustering we employed the louvain clustering algorithm with a resolution of 2.2 to obtain 11 subclusters. Marker genes between these subclusters were calculated with Seurat’s *FindMarker* function using default parameters.

#### Indirect calorimetry.

For indirect calorimetry, metabolic parameters of male and female POMC^Dre^ Drd2^Cre^ R26-lx-rx-hM3Dq-ZsGreen mice and control littermates (*Drd2^Cre–/–^ POMC^Dre–/–^ R26-lx-rx-hM3Dq-ZsGreen^+/–^* and *Drd2^Cre+/–^ POMC^Dre–/–^ R26-lx-rx-hM3Dq-ZsGreen^+/–^* mice) between 12 and 17 weeks of age were assessed in a Promethion Sable System (Promethion, Sable Systems). One week prior to measurements, animals were single-housed for acclimatization in metabolic cages and were handled daily. On day of measurement the recording was started 1 hour before dark phase, and data were acquired over a course of 24 hours with ad libitum access to NCD and water. CNO was i.p. injected at concentration of 3 mg/kg in 0.9% saline both at beginning of measurement and after 4 hours. Raw data were analyzed using the software ExpeData v.1.9.22 and Sable Systems Macro Interpreter v2.38 (Promethion, Sable Systems) via an analysis script with 10-minute data binning.

#### Infrared thermography.

For infrared thermography, imaging of male and female POMC^Dre^ Drd2^Cre^ R26-lx-rx-hM3Dq-ZsGreen mice and control littermates (*Drd2^Cre–/–^ POMC^Dre–/–^ R26-lx-rx-hM3Dq-ZsGreen^+/–^*, *Drd2^Cre+/–^ POMC^Dre–/–^ R26-lx-rx-hM3Dq-ZsGreen^+/–^*, and *Drd2^Cre–/–^ POMC^Dre+/–^ R26-lx-rx-hM3Dq-ZsGreen^+/–^* mice) at 12–20 weeks of age was performed using an FLIR E6-XT camera (Teledyne FLIR, FLIR E6-XT) equipped with a biconvex zinc selenide close-up lens for focal distances of 10.16 cm (EPSYS invent, custom made). The thermography camera was operating with a thermal sensitivity of <0.06°C, an accuracy of ±2% or ±2°C, and an infrared resolution of 240 × 180 pixels. Starting 1 week prior to measurements, mice were handled daily and acclimatized to imaging procedure. Two days prior to measurements, fur was removed from the animals in the area overlying the BAT. Mice were single-housed without nesting and minimum bedding 1 hour and fasted immediately before beginning of thermal imaging. Ad libitum access to water was maintained.

CNO was i.p. injected at concentration of 3 mg/kg in 0.9% saline and thermal images acquired at time points 0, 1, 2, 3, 4, 6, and 8 hours after CNO injection. A total of 2–7 images were acquired and analyzed of BAT and tail of freely behaving mice. For thermal imaging of the eye, mice were shortly fixed in the neck to ensure optimal focal distance. Thermal images were analyzed using the software FLIR tools V.6.4.18039.1003 by assessing the maximum temperature within eye and BAT, while defining the average temperature in a size-defined spot measurement at the tail base.

Additional methods details are in Supplemental Methods.

### Statistics

For statistical analysis of electrophysiological experiments, please refer to *Electrophysiological measurements*. For analysis of bacTRAP data, please refer to *bacTRAP RNA-Seq analysis*.

All other data were statistically analyzed using the GraphPad Prism v.9.2.0 software. Given normal distribution and equal variance between groups, data was analyzed using 2-tailed Student’s *t* test or 2-way ANOVA/2-way mixed effects models with Holm-Šídák or Tukey’s post hoc multiple comparisons test. Pairing and repeated measurements were assigned where applicable. Data without normal distribution or equal variance were analyzed with 2-tailed Wilcoxon or Mann-Whitney rank-sum tests. Pairing was assigned where applicable.

If not stated otherwise, data are represented as mean ± SEM; individual replicates are shown as dot plots. *P* values less than 0.05 were considered statistically significant.

### Study approval

All animal procedures were conducted in compliance with protocols approved by the local authorities (Bezirksregierung Köln, Cologne, Germany). Permission for breeding and experiments on mice was issued by the Department for Environment and Consumer Protection - Veterinary Section, Cologne, North Rhine-Westphalia, Germany [(§11) 576.1.35.2.G 07/18, 84-02.04.2017.A058].

## Author contributions

JCB and P Kloppenburg conceived the study. IG, SC, NB, and FTW developed methodology. IG, SC, NB, RN, L Shen, TSH, AJD, and WC investigated. IG, SC, P Klemm, and L Steuernagel performed formal analysis. IG, SC, P Klemm, and L Steuernagel visualized data. IG and JCB wrote the original draft. IG was the project administrator. JCB, FTW, and P Kloppenburg provided resources.

## Supplementary Material

Supplemental data

## Version 1 11/08/2022

Electronic publication
