# Supplementary material for: Dopamine-inhibited POMCDrd2+ neurons in the ARC acutely regulate feeding and body temperature
Source: JCI Insight. 2022 Nov 8;7(21):e162753. doi: 10.1172/jci.insight.162753 (PMC9675440; doi:10.1172/jci.insight.162753)
Supplement: Supplemental data [file jciinsight-7-162753-s202.pdf]

Supplemental Figure S1

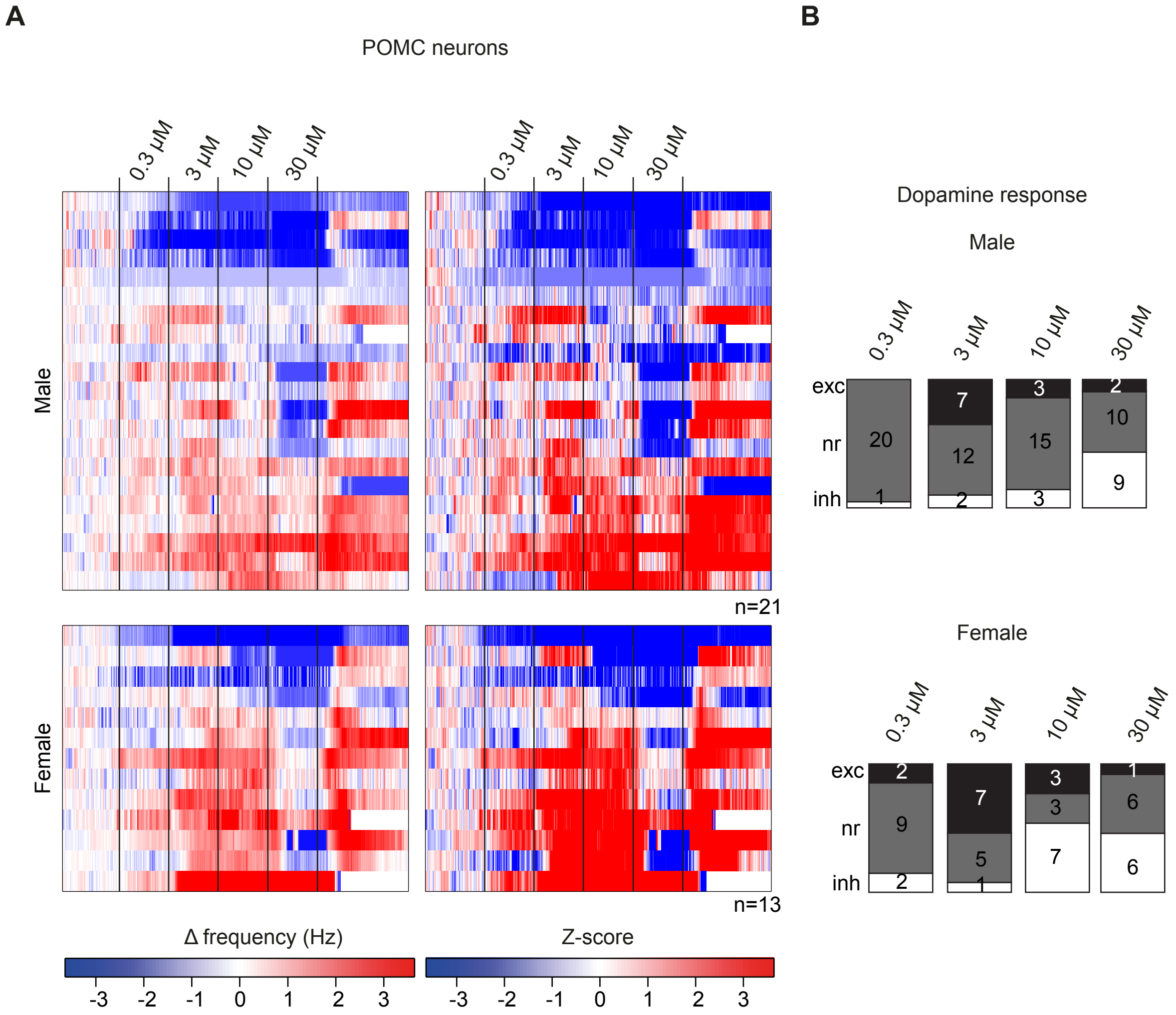

Supplemental Figure Titles and Legends

Supplemental Figure S1. Gender comparison of dopamine effect on POMC neurons.

(A) Heatmap of perforated patch-clamp recordings of POMC neurons in male (top) and female (bottom) $POMC^{GFP}$ mice between 11 and 20 weeks of age. Changes in action potential frequency from baseline and corresponding Z-scores during the application of increasing dopamine concentrations (0.3 μM, 3 μM, 10 μM, 30 μM) are depicted on the left and right, respectively. Data are also shown in Figure 3 (A) as merged dataset of both genders.

(B) Statistical qualification of dopamine responses in POMC neurons from male (top) and female (bottom) mice as quantified from (A) at indicated dopamine concentrations. A neuron was considered responsive, if the change in firing frequency induced by drug application was three times larger than the standard deviation. exc = excited; nr = not responsive; inh= inhibited. Data are also shown in Figure 3 (E) as merged dataset of both genders.

Supplemental Figure S2

A

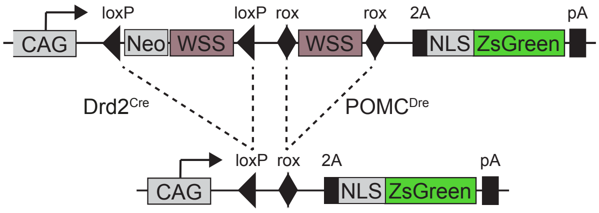

B

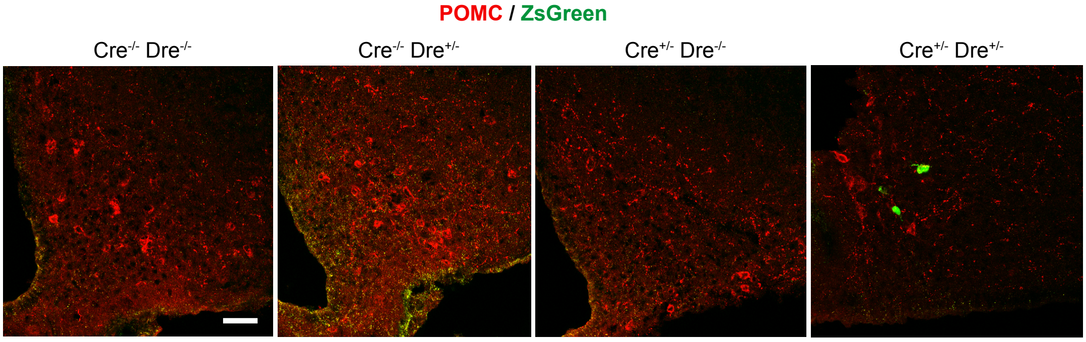

Supplemental Figure S2. Intersectional targeting specifically targets POMC^{Drd2+} neuronal subpopulation.

(A) Schematic illustration of genetic targeting strategy. Intersectional expression of Dre and Cre recombinase in *Drd2*-positive POMC neurons removes loxP- and rox-flanked stop cassettes in R26-lx-rx-ZsGreen mice, to allow for fluorescent reporter expression. (B) Representative images of immunofluorescent stainings against POMC and ZsGreen in ARC of all possible genotypes resulting from combinatorial Cre and Dre expression as depicted in (A). Scale bar represents 50 µm.

Supplemental Figure S3

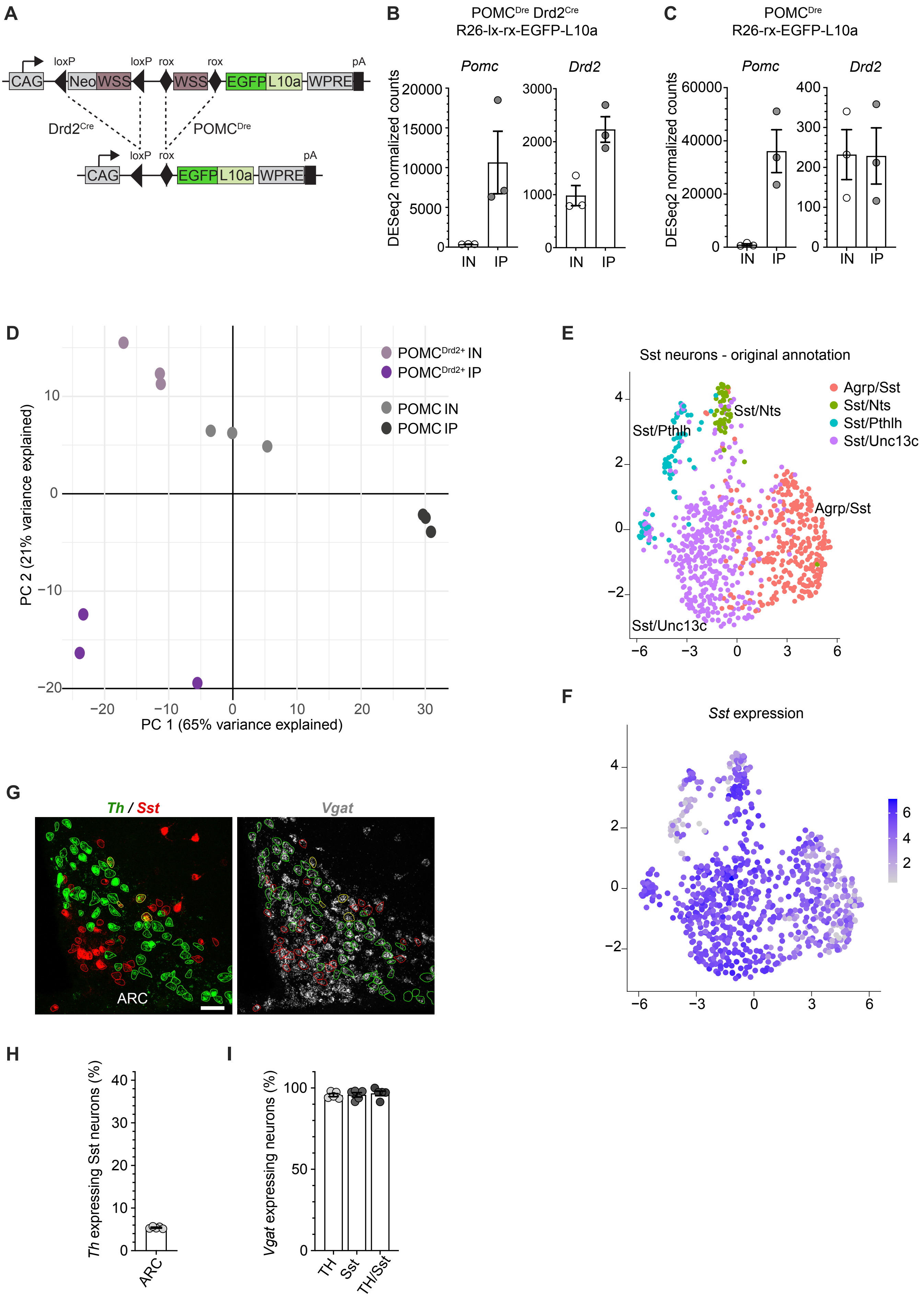

Supplemental Figure S3. Quality control of bacTRAP experiment.

(A) Schematic illustration of genetic targeting strategy. Intersectional expression of Dre and Cre recombinase in *Drd2*-positive POMC neurons removes loxP- and rox-flanked stop cassettes in R26-lx-rx-EGFP-L10a mice, to allow for transgene (EGFP-L10a) expression.

(B,C) Expression levels of *Pomc* (left) and *Drd2* (right) in bacTRAP IPs and inputs of $POMC^{Dre}$ $Drd2^{Cre}$ R26-lx-rx-EGFP-L10a (B) or $POMC^{Dre}$ R26-rx-EGFP-L10a mice (C). Differential gene expression analysis was performed using the DESeq2 1.28.0 (62) R package. Data are represented as mean ± SEM, n=3.

(D) Principal component analysis of bacTRAP IPs and inputs of $POMC^{Dre}$ $Drd2^{Cre}$ R26-lx-rx-EGFP-L10a and $POMC^{Dre}$ R26-rx-EGFP-L10a mice. n=3.

(E, F) UMAP plots showing clustering of ARC Sst clusters in single cell RNA sequencing data as defined by Campbell et al. (2) (E), and respective *Sst* mRNA expression levels (F).

(G) Representative images of RNA in situ hybridization against *Th* and *Sst* (left) and *Vgat* (right) in ARC of C57BL/6N mice. Scale bar represents 50 µm.

(H,I) Percentages of mediobasal hypothalamic *Th* co-expressing Sst neurons (H) or GABAergic TH and/or Sst neurons (I) as quantified from RNA in situ hybridization (G). No statistical tests were applied. Data are represented as mean ± SEM, n=5 per group.

Supplemental Figure S4

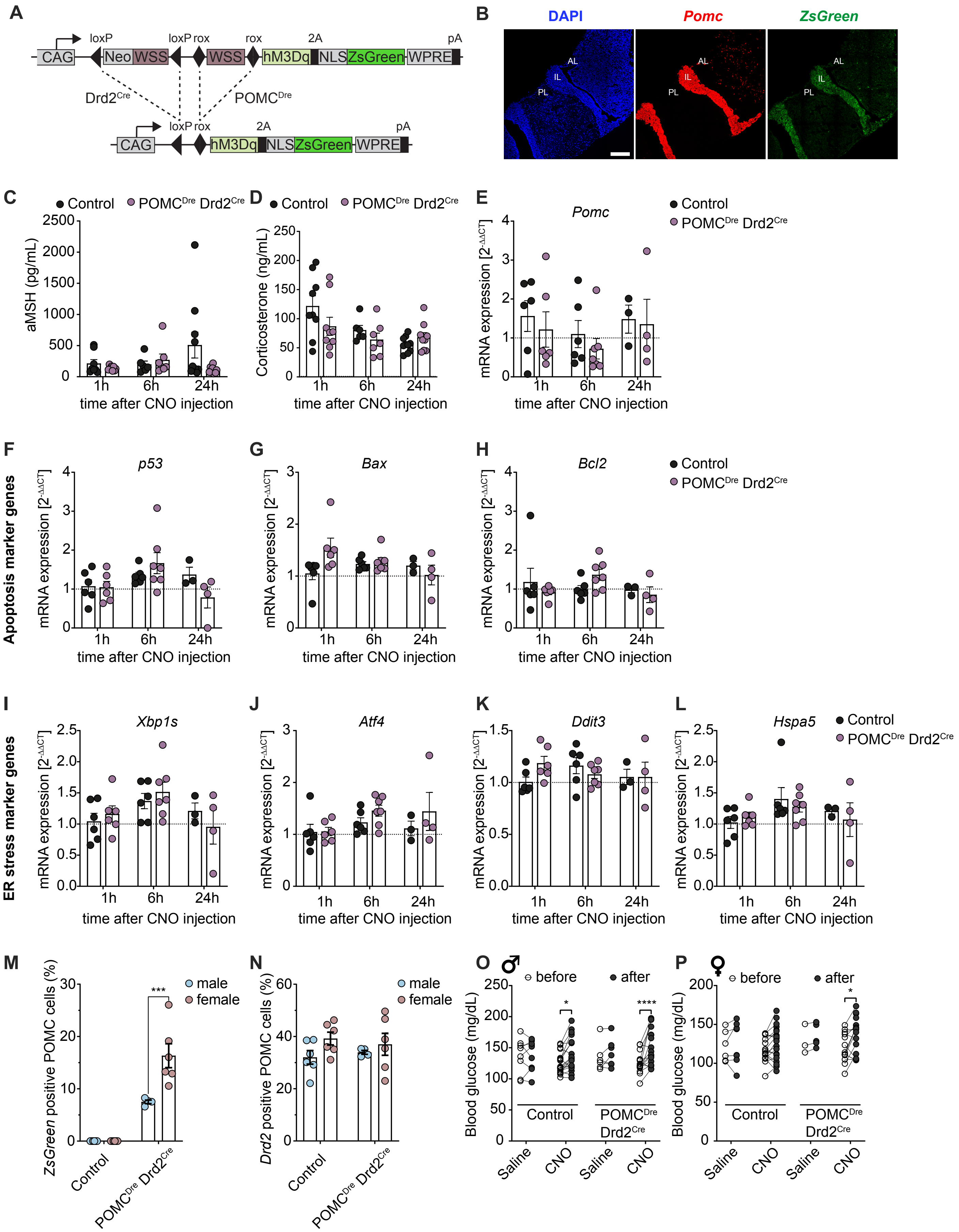

Supplemental Figure S4. Phenotyping of pituitary gland and circulating glucose levels in POMC^{Dre} Drd2^{Cre} R26-lx-rx-hM3Dq-ZsGreen mice.

(A) Schematic illustration of genetic targeting strategy. Intersectional expression of Dre and Cre recombinase in *Drd2*-positive POMC neurons removes loxP- and rox-flanked stop cassettes in R26-lx-rx-hM3Dq-ZsGreen mice, to allow for transgene (hM3Dq-ZsGreen) expression.

(B) Representative image of RNA in situ hybridization against *Pomc* and *ZsGreen* in pituitary gland of POMC^{Dre} Drd2^{Cre} R26-lx-rx-hM3Dq-ZsGreen mice. Scale bar represents 200 µm.

(C,D) Longitudinal αMSH (C) or corticosterone (D) levels in sera of CNO-injected POMC^{Dre} Drd2^{Cre} R26-lx-rx-hM3Dq-ZsGreen mice and control littermates (*Drd2*$^{Cre-/-}$ *POMC*$^{Dre-/-}$ *R26-lx-rx-hM3Dq-ZsGreen*$^{+/-}$ and *Drd2*$^{Cre+/-}$ *POMC*$^{Dre-/-}$ *R26-lx-rx-hM3Dq-ZsGreen*$^{+/-}$ mice). Data are represented as mean ± SEM, n=6-10 per group. *p*-values were calculated using unpaired two-tailed Student's t test per timepoint with Holm-Sidak's correction for multiple comparisons.

(E-L) Longitudinal mRNA expression levels of apoptosis and ER stress markers as indicated in pituitary glands of CNO-injected POMC^{Dre} Drd2^{Cre} R26-lx-rx-hM3Dq-ZsGreen mice and control littermates (*Drd2*$^{Cre-/-}$ *POMC*$^{Dre-/-}$ *R26-lx-rx-hM3Dq-ZsGreen*$^{+/-}$ and *Drd2*$^{Cre+/-}$ *POMC*$^{Dre-/-}$ *R26-lx-rx-hM3Dq-ZsGreen*$^{+/-}$ mice) as assessed by qPCR. Data are represented as mean ± SEM, n=3-7 per group. *p*-values were calculated using unpaired two-tailed Student's t test per timepoint with Holm-Sidak's correction for multiple comparisons.

(M,N) Quantification of RNA in situ hybridization (Figure 6A) reveals higher labelling efficiency in female than male POMC^{Dre} Drd2^{Cre} R26-lx-rx- hM3Dq-ZsGreen mice (M) despite balanced numbers *Drd2*-positive POMC neurons between genders (N). Controls are *Drd2*$^{Cre-/-}$ *POMC*$^{Dre-/-}$ *R26-lx-rx-hM3Dq-ZsGreen*$^{+/-}$ and *Drd2*$^{Cre+/-}$

POMC$^{Dre-/-}$ R26-lx-rx-hM3Dq-ZsGreen$^{+/-}$ littermates. Graph (M) re-uses data from Figure 6B, splitting it into male and female mice. Data are represented as mean ± SEM, n=4-6 per group. *p*-values were calculated using two-way ANOVA with with Sidak's post-hoc multiple comparisons test with a single pooled variance (gender).

(O,P) Blood glucose measurements before and 1 hour after saline or CNO injection in male (O) and female (P) POMC^{Dre} Drd2^{Cre} R26-lx-rx-hM3Dq-ZsGreen mice and control littermates (*Drd2$^{Cre-/-}$ POMC$^{Dre-/-}$ R26-lx-rx-hM3Dq-ZsGreen$^{+/-}$*, *Drd2$^{Cre+/-}$ POMC$^{Dre-/-}$ R26-lx-rx-hM3Dq-ZsGreen$^{+/-}$* and *Drd2$^{Cre-/-}$ POMC$^{Dre+/-}$ R26-lx-rx-hM3Dq-ZsGreen$^{+/-}$* mice). Data are represented as mean ± SEM, for male mice n=6-21 per group, for female mice n=3-20 per group. *p*-values were calculated using mixed-effects models with Sidak's post-hoc multiple comparisons test with a single pooled variance (time).

Supplemental Methods

Arcuate Nucleus micropunches

To obtain Arcuate Nucleus micropunch biopsies, 8 male WT C57BL/6N mice (Charles River, France) were decapited and brains quickly isolated. Fresh coronal brain sections of 300 µm thickness were cut in a stainless steel brain matrix (World Precision Instruments) and immediately frozen on dry ice. The mediobasal Arcuate Nucleus of the hypothalamus was consequently micro dissected from these frozen sections using a 1 mm diameter circular biopsy punch (Rainer Medizintechnik, Cat# KAI 101), samples snapfrozen in liquid nitrogen and stored at -80°C until RNA was isolated.

Total RNA isolation from tissue

For RNA isolation from bacTRAP samples, please refer to 'bacTRAP (bacterial artificial chromosome - translating ribosome affinity purification)'. For total RNA isolation from tissue, 1 mL Qiazol Lysis Reagent (QIAGEN, Cat# 79306) and 1.4 mm Zirconium oxide beads (Bertin Technologies, Cat# KT03961-1-103.BK) were given to the frozen tissues and samples were dissociated in a FastPrep-24™ homogenizer (MP Biomedicals, Fastprep-24 5G™) for 2 min at 30 Hz. 200 µL chloroform was given to the homogenates, samples vortexed vigorously for 15 sec and incubated for 3 min at room temperature. Samples were centrifuged for 15 min at 4°C and 12.000 rpm and upper aqueous phase mixed thoroughly with an even volume of chilled 70% EtOH in bidest. H_2O. RNA from Arcuate Nucleus micropunches of WT mice and from pituitary glands of POMC^{Dre} Drd2^{Cre} R26-lx-rx-hM3Dq-ZsGreen mice and control littermates (*Drd2*$^{Cre-/-}$ *POMC*$^{Dre-/-}$ *R26-lx-rx-hM3Dq-ZsGreen*$^{+/-}$ and *Drd2*$^{Cre+/-}$ *POMC*$^{Dre-/-}$ *R26-lx-rx-hM3Dq-ZsGreen*$^{+/-}$ mice) were subsequently extracted using the RNeasy Micro kit (QIAGEN, Cat# 74004) or RNeasy Mini kit (QIAGEN, Cat# 74104), respectively. RNA isolation protocols of the manufacturer were followed without alterations and without DNAse

treatment. RNA concentrations were determined by Nanodrop Spectrophotometer (Peqlab Biotechnologie, ND-1000).

Reverse transcription

Total RNA was reverse-transcribed using the High-Capacity cDNA Reverse Transcription Kit (Applied Biosytems, Cat# 4368813) according to manufacturer's instructions. 2 µL buffer, 4,2 µL of H 2O, 2 µL random hexamer primers, 1 µL RT enzyme and 0,8 mL dNTP per reaction were added to 10 µL total RNA. For cDNA synthesis of Arcuate Nucleus micropunches and pituitary glands, 85 ng or 1 µg RNA were used respectively. Reverse transcription was performed at 25°C for 10 min, at 37°C for 60 min and at 85°C for 5 min. After reverse transcription, cDNA of Arcuate Nucleus micropunches was used at resulting concentrations of 1,7 ng/µL, cDNA of pituitary glands was further diluted with 180 µL H_2O to a final concentration of 5 ng/µL.

Real time quantitative polymerase chain reaction (qPCR)

qPCR was performed using the TaqMan™ method by Applied Biosystems. In brief, 5 µL TaqMan™ Universal PCR Master Mix (Applied Biosystems, Cat# 4305719), 0,25 µL respective TaqMan™ assay, 0,75 µL H_2O and 4 µL of cDNA per reaction were mixed. cDNA of Arcuate Nucleus micropunches and cDNA of pituitary glands of $POMC^{Dre}$ $Drd2^{Cre}$ R26-lx-rx-hM3Dq-ZsGreen mice and control littermates (*$Drd2^{Cre-/-}$ $POMC^{Dre-/-}$ R26-lx-rx-hM3Dq-$ZsGreen^{+/-}$* and *$Drd2^{Cre+/-}$ $POMC^{Dre-/-}$ R26-lx-rx-hM3Dq-$ZsGreen^{+/-}$* mice) were used at concentrations of 1,7 ng/µL, cDNA or 5 ng/µL respectively. A list of all utilized TaqMan™ assays can be found in the 'Key Resources Table'. Hypoxanthine guanine phosphoribosyl transferase (*Hprt*) was used as housekeeping gene. qPCR amplification was performed using the QuantStudio 7 Flex Real-Time PCR System (Applied Biosystems, QuantStudio 7 Flex). qPCR

quantification was based on the ΔΔCT method, i.e. ΔCT values were normalized to the control group in the given experiment. Where no control group was applicable (Arcuate Nucleus micropunches of C57BL/6N mice) ΔCT values were normalized to the gene with the lowest expression within the experiment, i.e. *Drd4*.

Transcardial perfusion

Male WT C57BL/6N mice (Charles River, France) for RNA in situ hybridization were transcardially perfused at 12 weeks of age. Male and female POMC^{Dre} Drd2^{Cre} R26-lx-rx-hM3Dq-ZsGreen mice and control littermates (*Drd2*$^{Cre-/-}$ *POMC*$^{Dre-/-}$ *R26-lx-rx-hM3Dq-ZsGreen*$^{+/-}$, *Drd2*$^{Cre+/-}$ *POMC*$^{Dre-/-}$ *R26-lx-rx-hM3Dq-ZsGreen*$^{+/-}$ and *Drd2*$^{Cre-/-}$ *POMC*$^{Dre+/-}$ *R26-lx-rx-hM3Dq-ZsGreen*$^{+/-}$ mice) between 14-21 weeks of age were fasted at time point -120 min during the light cycle, *i.p.* injected with 3 mg/kg Clozapine N-oxide (CNO) in 0.9% saline at time point -60 min und trancardially perfused at time point 0 min. All mice were anesthetized with 800 mg/kg Avertin (2,2,2-Tribromoethanol, Sigma-Aldrich, Cat# T48402) in 0.9% saline and transcardially perfused with 0.9% saline followed by ice cold 4% paraformaldehyde (PFA; pH 7.4). Brains were removed and post-fixed in 4% PFA in 0.1 M phosphate buffered saline (PBS, pH 7.4) for 24 hours at 4°C. Samples were then incubated in 20 % sucrose in PBS for 12-24 hours at 4°C, frozen and stored at -80°C until sectioning.

Brain sectioning

PFA-fixed, frozen brain tissues were cut in a cryostat (Leica, CM3050S) at a chamber temperature of -20°C. For immunohistochemical analyses sections were cut at a thickness of 30 μm and mounted onto polysine-coated glass slides (ThermoFisher, Cat# J2800AMNZ). For RNA in situ hybridization sections were cut at a thickness of

20 µm and mounted onto SuperFrost Plus Gold slides (ThermoFisher, Cat#11976299). Slides were stored at -80°C until further processing.

Microscopy and image processing

Microscopic images of RNA in situ hybridization for *Sst/Th* overlap were obtained with an Olympus SLIDEVIEW VS200 digital slide scanner at a 20x magnification (objective: UPLXAPO 20x/0.8; software: VS200 ASW), all other microscopic images were acquired using a confocal Leica TCS SP-8-X microscope at 40x magnification and z-stack size of 0.9 µm (objective: 40x/1.30oil, software: LASX V.3.5.7.23225). Laser intensities were kept constant throughout all related conditions and adjustments in brightness and contrast of all channels, as depicted in the representative microscopic images, were applied equally throughout all related conditions.

Immunofluorescent stainings

For immunofluorescent stainings 14-16 week old male and female POMC^{Dre} Drd2^{Cre} R26-lx-rx-ZsGreen mice and 11-14 week old male and female POMC^{Dre} Drd2^{Cre} R26-lx-rx-EGFP-L10a mice were perfused and brain sections mounted as described above (‘Transcardial perfusion’ and ‘Brain sectioning’). For immunohistochemical assays, brain sections were thawed, post-fixed for 10 min in 4% PFA in PBS, washed twice for 10 min PBS (pH 7.4), incubated for 10 min in 0.3 % glycine in PBS, washed once as before, incubated for 10 min in 0.03 % SDS in PBS and incubated in blocking solution (3 % donkey serum in PBS containing 0.25% Triton X-100) for 60 minutes at room temperature. Primary antibody incubation took place over night at 4°C at the following dilutions: anti-ZsGreen (Takara Bio Clontech, Cat# 632474; RRID:AB_2491179) 1:100, anti-POMC (Phoenix Pharmaceuticals, Cat# H-029-30; RRID:AB_2307442) 1:1000 and anti-GFP (Abcam, Cat# ab13970; RRID:AB_300798) 1:1000. Slides were

washed 3 times for 10 min with washing buffer (PBS + 0.1% Triton X-100) and incubated with the respective secondary antibody for 60 minutes in the dark at room temperature. All utilized secondary antibodies are listed in the 'Resources Table' and were applied to samples at a dilution of 1:500. Samples were washed as before in washing buffer and mounted using Vectashield Antifade Mounting Medium with DAPI (Vector Laboratories). Co-stainings that utilized two antibodies from the same host (ZsGreen and POMC in brain sections of $POMC^{Dre}$ $Drd2^{Cre}$ R26-lx-rx-ZsGreen mice) were stained for each target protein on discrete days, separated by an additional 10 min fixation in 4 % PFA in PBS, PBS wash and 60 min incubation in blocking solution at room temperature after completed staining for first target protein including its secondary antibody. Specifically, in $POMC^{Dre}$ $Drd2^{Cre}$ R26-lx-rx-ZsGreen mice staining against ZsGreen was performed one day prior to staining against POMC. Microscopic images of immunofluorescent stainings in $POMC^{Dre}$ $Drd2^{Cre}$ R26-lx-rx-ZsGreen and $POMC^{Dre}$ $Drd2^{Cre}$ R26-lx-rx-EGFP-L10a mice were acquired using a confocal Leica TCS SP-8-X microscope ('Microscopy and image processing') and images analyzed utilizing the image processing software ImageJ (ImageJ 1.53f51) and its Cell Counter plugin (by Kurt De Vos, University of Sheffield).

Enzyme-linked immunosorbent assays (ELISAs)

For quantification of circulating αMSH and corticosterone levels, ELISAs were performed on sera of male and female $POMC^{Dre}$ $Drd2^{Cre}$ R26-lx-rx-hM3Dq-ZsGreen mice and control littermates ($Drd2^{Cre-/-}$ $POMC^{Dre-/-}$ *R26-lx-rx-hM3Dq-ZsGreen*$^{+/-}$ and $Drd2^{Cre+/-}$ $POMC^{Dre-/-}$ *R26-lx-rx-hM3Dq-ZsGreen*$^{+/-}$ mice) between 14-20 weeks of age. Mice were *i.p.* injected with 3 mg/kg in 0.9% saline at 24 hours, 6 hours or 1 hour prior to sacrifice. Mice were decapitated, blood collected and incubated for at least 30 min on ice to allow clotting. Samples were centrifuged for 20 min at 10,000 rpm and 4°C,

supernatants snapfrozen in liquid nitrogen and stored at -80°C until further processing. αMSH levels were determined utilizing the mouse αMSH ELISA Kit (Abbexa, Cat#abx254513) according to manufacturer's instructions with following specifications: samples were processed in 1:2 dilutions and TMB substrate incubation was set to 20 min. Corticosterone levels were determined utilizing the mouse Corticosterone ELISA Kit (Crystal Chem, Cat#80556) according to manufacturer's instructions without modifications. Optical densities of standards and samples were assessed using a FilterMax F5 Multi-Mode microplate reader and SoftMax Pro 6.3 software (Molecular Devices).

Blood glucose measurements

Blood glucose levels were determined in male and female POMC^{Dre} Drd2^{Cre} R26-lx-rx-hM3Dq-ZsGreen mice and control littermates (*Drd2*$^{Cre-/-}$ *POMC*$^{Dre-/-}$ *R26-lx-rx-hM3Dq-ZsGreen*$^{+/-}$, *Drd2*$^{Cre+/-}$ *POMC*$^{Dre-/-}$ *R26-lx-rx-hM3Dq-ZsGreen*$^{+/-}$ and *Drd2*$^{Cre-/-}$ *POMC*$^{Dre+/-}$ *R26-lx-rx-hM3Dq-ZsGreen*$^{+/-}$ mice) between 12-18 weeks of age. Mice were fasted at time point -120 min during the light cycle and *i.p.* injected with either 0.9% saline or 3 mg/kg Clozapine N-oxide (CNO) in 0.9% saline at time point -60 min. Immediately before and one hour after CNO injection blood glucose levels were assessed on blood from a microincision in the tail tip using a Contour glucometer (Bayer).

Supplemental Tables

RESOURCES TABLE

REAGENT or RESOURCE	SOURCE	IDENTIFIER
**Antibodies**		
Rabbit polyclonal anti-ZsGreen	Takara Bio Clontech	Cat# 632474; RRID:AB_2491179
Rabbit polyclonal anti-POMC	Phoenix Pharmaceuticals	Cat# H-029-30; RRID:AB_2307442
Chicken polyclonal anti-GFP	Abcam	Cat# ab13970; RRID:AB_300798
Heintz Lab TRAP anti-GFP 19C8 antibody	Heintz Lab; Rockefeller University	Cat# Htz-GFP-19C8; RRID:AB_2716737
Heintz Lab TRAP anti-GFP 19F7 antibody	Heintz Lab; Rockefeller University	Cat# Htz-GFP-19F7; RRID:AB_2716736
Donkey anti-Rabbit polyclonal Secondary Antibody, Alexa Fluor 488	Invitrogen	Cat# A-21206; RRID:AB_2535792
Donkey anti-Rabbit polyclonal Secondary Antibody, Alexa Fluor 594	Invitrogen	Cat# A-21207; RRID:AB_141637
Goat anti-Rabbit polyclonal Secondary Antibody, Alexa Fluor 594	Invitrogen	Cat# A-11012; RRID:AB_141359
Goat anti-Chicken polyclonal Secondary Antibody, FITC	Jackson ImmunoResearch Labs	Cat# 103-095-155; RRID:AB_2337384
**Chemicals, peptides, and recombinant proteins**		
Dopamine hydrochloride	Sigma-Aldrich	Cat#H8502
Quinpirole hydrochloride	Sigma-Aldrich	Cat#Q102
Somatostatin	Sigma-Aldrich	Cat#S1763
HEPES	AppliChem	Cat# A1069,0250
Nonident P40	AppliChem	Cat# A1694,0250
DTT	AppliChem	Cat# A1101,0005
Cycloheximide	AppliChem	Cat# A0879,0001
Complete Mini, EDTA-free	Sigma-Aldrich	Cat#11836170001
PhosSTOP (Phosphatase Inhibitor Cocktail)	Sigma-Aldrich	Cat#04906845001
RNasin	Promega	Cat# N2511
DHPC (1,2-diheptanoyl-sn-glycero3-phosphocholine)	Avanti Polar Lipids	Cat#850306P
Qiazol Lysis Reagent	QIAGEN	Cat# 79306
Clozapine N-oxide	Abcam	Cat# ab141704
Glucose, 20 % solution	B. Braun	N/A
**Critical commercial assays**		
TaqMan® Gene Expression Assay Drd1	Applied Biosystems	Cat# Mm01353211_m1
TaqMan® Gene Expression Assay Drd2	Applied Biosystems	Cat# Mm00438545_m1
TaqMan® Gene Expression Assay Drd3	Applied Biosystems	Cat# Mm00432887_m1
TaqMan® Gene Expression Assay Drd4	Applied Biosystems	Cat# Mm00432893_m1

TaqMan® Gene Expression Assay Drd5	Applied Biosystems	Cat# Mm00658653_s1
TaqMan® Gene Expression Assay Hprt	Applied Biosystems	Cat# Mm01545399_m1
TaqMan® Gene Expression Assay Pomc	Applied Biosystems	Cat# Mm00435874_m1
TaqMan® Gene Expression Assay Trp53	Applied Biosystems	Cat#Mm01731287_m1
TaqMan® Gene Expression Assay Bax	Applied Biosystems	Cat# Mm00432050_m1
TaqMan® Gene Expression Assay Bcl2	Applied Biosystems	Cat# Mm00477631_m1
TaqMan® Gene Expression Assay Xbp1s (spliced)	Applied Biosystems	Cat#Mm03464496_m1
TaqMan® Gene Expression Assay Atf4	Applied Biosystems	Cat#Mm00515324_m1
TaqMan® Gene Expression Assay Ddit3	Applied Biosystems	Cat# Mm00492097_m1
TaqMan® Gene Expression Assay Hspa5	Applied Biosystems	Cat# Mm00517691_m1
2,2,2-Tribromoethanol	Sigma-Aldrich	Cat# T48402
RNAscope® Probe- Mm-Pomc	Advanced Cell Diagnostics	Cat#314081
RNAscope® Probe- Mm-Agrp	Advanced Cell Diagnostics	Cat# 400711
RNAscope® Probe- Mm-Drd1	Advanced Cell Diagnostics	Cat# 406491
RNAscope® Probe- Mm-Drd2	Advanced Cell Diagnostics	Cat# 406501
RNAscope® Probe- Mm-Lepr-tv1	Advanced Cell Diagnostics	Cat# 471171
RNAscope® Probe- Mm-Glp1r	Advanced Cell Diagnostics	Cat# 418851
RNAscope® Probe- Mm-Th	Advanced Cell Diagnostics	Cat# 317621
RNAscope® Probe- Mm-Sstr1	Advanced Cell Diagnostics	Cat# 437711
RNAscope® Probe- Mm-Fos	Advanced Cell Diagnostics	Cat# 316921
RNAscope® Probe- Mm-ZsGreen	Advanced Cell Diagnostics	Cat#461251
RNAscope® Probe- Mm-Sst	Advanced Cell Diagnostics	Cat#404631
RNAscope® Fluorescent Multiplex Detection Reagents v2	Advanced Cell Diagnostics	Cat#323100
RNAscope® 4-Plex Ancillary Kit for Multiplex Fluorescent Kit v2	Advanced Cell Diagnostics	Cat# 323120
Probe diluent for RNAscope®	Advanced Cell Diagnostics	Cat# 300041
Mouse Alpha-Melanocyte Stimulating Hormone (αMSH) ELISA Kit	Abbexa	Cat# abx254513
Mouse Corticosterone ELISA Kit	Crystal Chem	Cat# 80556
RNeasy Mini kit	QIAGEN	Cat#74104
RNeasy Micro kit	QIAGEN	Cat#74004
Takyon™ Low ROX Probe 2X MasterMix dTTP blue	Eurogentec	Cat# UF-LPMT-B0701
High-Capacity cDNA Reverse Transcription Kit	Applied Biosytems	Cat# 4368813

**Deposited data**		
POMC^{Drd2} BacTRAP RNA Seq data	This paper	GSE210311
POMC BacTRAP RNA Seq data	This paper	GSE210311
**Software and algorithms**		
ImageJ 1.53f51	ImageJ	
FLIR tools V.6.4.18039.1003	Teledyne FLIR	
VS200 ASW	Olympus	
GraphPad Prism v.9.2.0	GraphPad Software	
ExpeData v.1.9.22	Sable Systems	
Sable Systems Macro Interpreter v2.38	Sable Systems	
**Other**		
Normal chow diet (NCD)	ssniff Spezialdiäten	Cat# V1554-703
Confocal microscope	Leica	Leica TCS SP-8-X
Slidescanner microscope	Olympus	SLIDEVIEW VS200
Protein A Dynabeads	Invitrogen	Cat# 10001
FLIR thermal camera	Teledyne FLIR	FLIR E6-XT
Zinc-selenide lense, focal distance 10.16 cm	Epsys invent	N/A
QuantStudio 7 Flex Real-Time PCR system	Applied Biosystems	QuantStudio 7 Flex
Microplate reader	Molecular Devices	FilterMax F5 Multi-Mode Microplate Reader
Sable system	Promethion	
SuperFrost Plus Gold slides	ThermoFisher	Cat# K5800AMNT72
Vectashield Antifade Mounting Medium with DAPI	Vector Laboratories	Cat# H-1200
